# Genome-wide DNA-(de)methylation is associated with Noninfectious Bud-failure exhibition in Almond (*Prunus dulcis* [Mill.] D.A.Webb)

**DOI:** 10.1038/srep42686

**Published:** 2017-02-16

**Authors:** Jonathan Fresnedo-Ramírez, Helen M. Chan, Dan E. Parfitt, Carlos H. Crisosto, Thomas M. Gradziel

**Affiliations:** 1 Department of Plant Sciences, University of California, Davis, CA 95616, USA

## Abstract

Noninfectious bud-failure (BF) remains a major threat to almond production in California, particularly with the recent rapid expansion of acreage and as more intensive cultural practices and modern cultivars are adopted. BF has been shown to be inherited in both vegetative and sexual progeny, with exhibition related to the age and propagation history of scion clonal sources. These characteristics suggest an epigenetic influence, such as the loss of juvenility mediated by DNA-(de)methylation. Various degrees of BF have been reported among cultivars as well as within sources of clonal propagation of the same cultivar. Genome-wide methylation profiles for different clones within almond genotypes were developed to examine their association with BF levels and association with the chronological time from initial propagation. The degree of BF exhibition was found to be associated with DNA-(de)methylation and clonal age, which suggests that epigenetic changes associated with ageing may be involved in the differential exhibition of BF within and among almond clones. Research is needed to investigate the potential of DNA-(de)methylation status as a predictor for BF as well as for effective strategies to improve clonal selection against age related deterioration. This is the first report of an epigenetic-related disorder threatening a major tree crop.

Noninfectious bud-failure (BF) is a disorder in almond (*Prunus dulcis* [Mill.] D.A.Webb), expressed as a failure of vegetative bud development leading ultimately to tree decline and is not associated with any pathogenic organisms^1^. BF is a particularly serious problem for the commercially important cultivars ‘Nonpareil’ and ‘Carmel’, which together make up over 50% of total almond production in California. This is due, in part, to the fact that these cultivars have been clonally propagated for a longer period than other more recently developed cultivars. Careful clonal source selection for low BF-potential has allowed for continued plantings of both ‘Nonpareil’ and ‘Carmel’ after BF first became a serious problem in these cultivars. BF-potential appears to be related to the tree age as well as the clone age and cumulative exposure to high temperature during multiple propagation cycles of the cultivar^2^. BF may not be sufficiently low in even the best clonal sources of ‘Carmel’ to ensure continued commercial plantings. Careful selection of low-BF ‘Nonpareil’ clones in the 1970s, 80 s and 90 s has allowed for continued plantings of this dominant variety, though recent observation of BF-exhibition in some elite ‘Nonpareil’ clone sources indicate that they may also be progressing towards BF-exhibition. High levels of BF-exhibition have been a major contributor to the early abandonment of many otherwise promising cultivars, including ‘Merced’ and ‘Jordanolo’, and will likely be found in some of the recently released California varieties, particularly those which have the BF-susceptible cultivar ‘Nonpareil’ as a parent. While BF has been shown to be transferred to both vegetative and sexual progeny, knowledge of a mechanism for the genetic control remains elusive.

Previous research suggests that BF is a result of the failure of gene/gene complex expression required for normal winter dormancy and associated development of the vegetative bud^2^. This failure may be promoted by an epigenetic-like mechanism triggered by heat stress during the previous growing season^1^,^2^,^3^. DNA fragment bands in agarose gel electrophoresis appear to be identical in both the low and high exhibition BF clones of the same cultivar, obviating the value of traditional DNA-based markers for analyzing this disorder, particularly with the absence of a publicly available assembled and annotated genome sequence for almond. Similar epigenetic-like disorders are relatively common in other long propagated *Prunus* sp. clones including ‘Rusty-Blotch’ and ‘False Shot-Hole’ disease in plums, ‘Russet-Scab’ in prune, ‘Gumboil’ in apricot, and ‘Crinkle-Leaf’ disease in cherry and plum^3^. However, the atypical, epigenetic-like nature of BF and similar disorders has not been explained by previous genetic and genomic research.

Recent advances in our understanding of whole plant genomics has begun to elucidate the diversity of epigenetic mechanisms playing important roles in plant development as well as ageing related changes in many diverse organisms^4^,^5^,^6^,^7^,^8^,^9^,^10^. DNA methylation is a widely reported epigenetic mechanism involving a biochemical process in which a methyl group is added to the 5′ position of the cytosine pyrimidine ring or the number 6 nitrogen of the adenine purine ring in the adenine (A) or cytosine (C). The presence and absence of methylation in the DNA [henceforth, DNA-(de)methylation] affect clonal (mitotic) and seedling (meiotic) inheritance. This phenomenon is associated with gene silencing during development and ageing in higher organisms, including the angiosperms^6^. Examples include vernalization in Crucifers^11^ and cereals^12^, as well as induction to flowering in species such as sugar beet^13^.

Previous studies have led to an understanding of the pattern of BF development within clones, allowing for the effective selection of clonal sources whose vegetative progeny are less likely to express BF during the crucial early years of scion development and entering the commercial productive stage^2^,^14^,^15^. Attempts to elucidate the inheritance of BF-potential suggest that the likelihood of progenies from parents exhibiting BF is proportional to the BF-potential of the parents^16^,^17^,^18^. Kester *et al*.^2^ also reported that BF-exhibition of twelve unique nursery sources was distinct in separation and dynamics patterns corresponding with the clonal age of the source, which suggests somaclonal variation not explained by DNA polymorphisms itself, presumably because the disorder is not associated with changes in the DNA sequence of the controlling gene(s), but rather involving the modification of gene activity through still poorly understood epigenetic-like mechanisms^19^,^20^. Similarly, all attempts to identify genetic polymorphisms linked with BF-potential have been unsuccessful. In addition, it has been suggested that different types of BF may exist. Thus ‘Nonpareil’ and cultivars having it in their pedigree, exhibit one related type of BF, while other unrelated cultivars such as ‘Mission’ (syn. ‘Texas’), ‘Peerless’, ‘Ai’, ‘Rana’, ‘Marcona’, ‘Dobkov’ exhibit another type of BF, which may be exhibited in a similar way but are caused by different mechanisms^14^.

As part of this research, the Almond Breeding Program at UC Davis collected and characterized diverse clonal sources of different cultivars, which vary in their potential for exhibiting BF. DNA-(de)methylation has been assessed in this material through Methylation profiles using methylation-sensitive amplified fragment length polymorphism (MS-AFLPs) in order to test for associations with increasing potential for BF-exhibition as well as ageing.

## Methods

### Almond leaf samples and data collection

Almond leaf samples from clones differing in BF-exhibition were collected from six diverse almond propagation sources, including samples from individual clonal trees, which had been documented to be over 10 decades (>100 years) old (Table 1). For each accession, a numerical score of 6, 5, 4, 3, 2 or 1 (for high, medium to high, medium, low to medium, low and no bud failure exhibition) was assigned, as well as the estimated clonal age (time in decades since a specific clone was initially used as a budwood source for propagation, based on nursery records).

‘Nonpareil’ is the dominant commercial cultivar in California, accounting for approximately 40% of total plantings with the remaining cultivars primarily planted as its pollinizers^21^. Nonpareil, along with ‘Mission’, originated as seedling selections in the mid-1800’s and became the basis for modern commercial production, including serving as parents to most of the current pollinizer cultivars. Because individual almond trees can live for 100 years or more, trees from these early plantings are still available for sampling. Despite extensive plantings over the past 150 years, BF-exhibition in the cultivar ‘Mission’ has only been identified in a few propagation sources. ‘Carmel’, which originated as a ‘Mission’ × ‘Nonpareil’ seedling in the mid-1900’s, had become the second most important commercial cultivar until high levels of BF reduced its planting to approximately 10% of current acreage. ‘Turkmen’ is an almond selection from Turkmenistan where high BF-exhibition was observed in trees propagated from terminal buds of the original tree while low BF-exhibition was observed in trees propagated from basal buds. Stukey-5–1 and Stukey-5-2 were propagated from a single polyembryonic seed derived from early Nonpareil seed embryo budding and so are genetically identical^22^, except that the tree from Stukey-5-2 displayed BF symptoms after 3 years while Stukey-5-1 remains free of BF after over 12 years. Stukey-6-1 and Stukey-6-2 also were derived from similar ‘Nonpareil’ polyembryonic seed, also showing similar divergent BF development patterns. The ‘Winters’ cultivar was released in the late 1990’s and despite extensive planting has displayed BF in only a few individual trees from a single propagation source.

### Almond leaf genomic DNA isolation

Almond leaf genomic DNA was isolated following the methods of Doyle and Doyle^23^ and Keb-Llanes^24^. The genomic DNA samples were further purified and concentrated through the use of the Qiagen DNeasy Plant Mini kit (QIAGEN Inc., Valencia, CA, USA), according to the manufacturer’s instructions. The quality of the isolated almond leaf genomic DNA was visualized through electroporation of the DNA samples in a 1% agarose gel at 50 V for 1 hour. The DNA samples were subjected to spectrophotometric analysis with the Eppendorf BioPhotometer Plus (Eppendorf, Hamburg, Germany) to obtain isolated DNA. All genomic DNA samples were diluted to 300 ng/μl for use in the AFLP analysis.

### MS-AFLP Analysis

The methylation-sensitive amplified fragment length polymorphisms protocol involves the use of the isoschizomers *Hpa*II and *Msp*I. Adapters for *Hpa*II-*Msp*I were designed through the annealing of the oligonucleotides 5′-GATCATGAGTCCTGCT-3′ and 3′-AGTACTCAGGACG AGC-5′, with a 5′ GA and GC overhang. The details of the protocol are provided in Supplementary File.pdf page 2 and in Tables S1 and S2.

### Data analysis

The data from the MS-AFLP analysis were recorded in a comma separated (csv) file, to be read in the library MSAP ver. 1.6.3 [ref. 25] for the statistical language R ver. 3.0.1 [ref. 26]. The binary matrix was configured following the MSAP ver. 1.6.3 convention, a first column indicating the category of BF-exhibition, followed by a column indicating the name of the accession, a column for the correspondent fragment (*Eco*RI or *Hpa*II/*Msp*I) with the following columns being the primer combination, in accordance with the order of scoring (as shown in Table S3) composed of the primer (from *a* to *i*) and the corresponding scoring band. For BF-exhibition levels, a scale based on six categories as described above was used. Additionally, models considering dichotomous outcomes (presence/absence) for BF-exhibition were performed in order to contrast the results yielded with models considering polytomous outcomes.

In the study of the causal relationships between MS-AFLP bands and BF-exhibition, only the distinct, reproducible, well-resolved MS-AFLP fragments scored as present (1) or absent (0), together with the data for the scoring of BF, were used for the construction of a matrix for the development of a dendrogram of DNA methylation based relationships. Subsequently, conclusions were drawn about the clustering of the sampled accessions according to the covariance of methylations status profiles and BF-exhibition.

Matrices from Principal Coordinates Analysis from the application of the package MSAP, were used to generate a heatmap for visual evaluation of the association of the (de)methylated sites with BF-exhibition among accessions. The assessment of similarity of (de)methylation patterns between and within genotypes and clones was performed through the Kendall’s tau (τ) coefficient adjusted for ties for the number of concordant and discordant pairs for presence absence of a band per primer combination. Subsequent conditional variable independence tests through contingency tables using the packages vcd^27^ and VCDextra^28^ were performed to assess the association between methylation and BF-exhibition, as illustrated using mosaic plots.

Finally, a Bayesian analysis of complete contingency tables through hierarchical log-linear model selection was performed using the R package Conting^29^ in order to assess the independence of DNA-(de)methylation status with respect to BF-exhibition, clonal age and almond genotype.

The function bcct was used for model selection with consideration up to the saturated model, and which includes the triple interaction (DNA-(de)methylation status × clonal age × almond genotype). The model was introduced as follows: *BudFailureLN~(deMet01* + *ChronoAge* + *GenotypeN*)^*3*^, as indicated by the user manual of Conting. *BudFailureLN* is the numerical score of BF-exhibition recorded for the clone, which was assessed as a polytomous response. *deMet01* is the binary score expressed as 0 and 1 for presence/absence of methylation in the DNA per band and was considered as a factor, *ChronoAge* refers to the time in decades since a specific clone was initially used as a propagation source and is a continuous variable in the model. *GenotypeN* is the numerical ID for each almond genotype, also used as factor. The Monte Carlo Markov Chain (MCMC) iterations used to ensure convergence were 12,500. Inferences were made using 10,000 effective iterations after a burn-in of the first 2500 iterations, without thinning of the chains as recommended by Link and Eaton^30^.

The cutoff for significance was 0.05 for the report of the best three models according to the deviance statistic calculated from the posterior distribution. The prior used for this analysis was the generalized hyper-g prior proposed by Sabanés-Bové and Held^31^ implemented as default prior for analysis of contingency in tables in Conting. The adequacy of the model was evaluated through Bayesian (or posterior predictive) *p*-value (*p*_*B*_) using the Deviance statistic^32^ as the hypothesis test, which basically is −2 times the log-likelihood ratio of the independence model (model without interactions) compared to the saturated model (the model including double and triple interactions).

Model selection was also performed for the clonal accessions of ‘Nonpareil’ (nine clones), following the same procedure described above using model selection with consideration up to the saturated model, which included the interaction between DNA-(de)methylation status × clonal age, introduced as: *BudFailureNP~(deMet01* + *ChronoAge*)^*2*^. Calculation of log-odds ratios and probabilities for the parameters in the final model were performed by fitting the model with the function glm and the routines available in the statistics base of R 3.0.1. To address the goodness of fit and estimate the proportion of the variance explained for the selected models, the *adjusted pseudo-R*^*2*^ of McFadden^33^ (which is considered a likelihood-ratio index) was used for models including all the genotypes evaluated, as well as the ‘Nonpareil’ clones, considered separately.

## Results

### Methylation-sensitive amplified fragment length polymorphisms analysis

Once the cultivar identity was confirmed for every clone through AFLP analysis, a total of 1251 fragments were scored in nine primer combinations per accession. 24,794 bands were scored in total for 22 accessions (LI-COR images in Supplementary File pages 5 to 23). The number of scored bands in the primer combination *a* (Table S3) was 120, 136 for the primer combination *b,* 125 for the primer combination *c*, 161 for the primer combination *d*, 114 for the primer combination *e*, 214 for the primer combination *f*, 133 for the primer combination *g*, 121 for the primer combination *h*, and finally 124 for the primer combination *i* (Supporting data.xls).

The banding pattern of 1238 fragments showed evidence for loci susceptible to methylation (Methylation-Susceptible Loci), of which 1129 fragments were polymorphic (91%). The methylation fractions (as a function of the number of evaluated bands) associated with accessions exhibiting BF (*BF* accessions) and accessions non-exhibiting BF (*NBF* accessions) associated with specific primer combination types are shown in Table 2. Thus, of the 24,794 bands, 6598 (26.61%) represented methylated loci and 5799 (23.39%) represented un-methylated loci in *BF* accessions, while 5798 (23.38%) represented methylated loci and 6597 (26.60%) represented un-methylated loci in *NBF* accessions. This data provided information for a first approach to test for independence between BF-exhibition and DNA-(de)methylation, which is shown as a mosaic plot in Figure S1. The magnitude of the Pearson residuals (the individual contribution to the Pearson χ^2^ statistic for independence of binomial distribution) the *p*-value (<2.22e^−16^), and correlation (0.064) suggest a departure from independence, i.e. DNA-(de)methylations is not an independent event with respect BF-exhibition. The bands *e*_22_ and *i*_19_ (the order of the band goes from heavy to light fragment) demonstrated total correspondence with BF-exhibition (presence for *BF* accessions and absence for *NBF* accessions). These bands correspond to the primer combinations: E-AGT + HM-TAA and E-ACG + HM-TAA, respectively.

**Figure 1:**
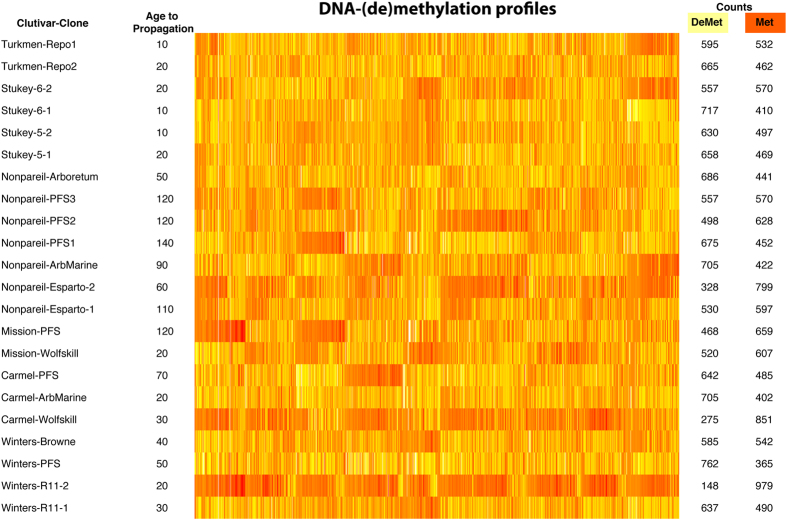
Heatmap for visual inspection. The age from initial propagation is the approximated chronological years (10 years = 1 decade) since the accession was first used as a source for clonal propagation. The counts include demethylated (DeMet, yellow cells) and methylated (Met, orange cells) sites.

The analysis of the three-way contingency table for assessment for independence of BF-exhibition, DNA-(de)methylation and clonal age was primarily assessed through the visual inspection of a heatmap (Fig. 1) and supported by the Kendall τ correlation coefficients to assess the concordance in patterns of DNA-(de)methylation. The heatmap suggested differential banding pattern among and within clones. Amongst cultivars, Kendall τ averaged around 0.29, indicating positive significant concordance, although low, suggesting differentiated DNA-(de)methylation among almond genotypes. Kendall τ ranged from 0.10 for the concordance between ‘Mission’ and ‘Carmel’ to 0.55 between Stukey-5 and Stukey-6 (Table S4). The concordance among clones showed variations from 0.07 concordance between Nonpareil_PFS2 and Winters_R11_2, to 0.61 between Winters_Browne and Winters_R11_1, with an average of 0.18 among clones (Table S5).

**Figure 2:**
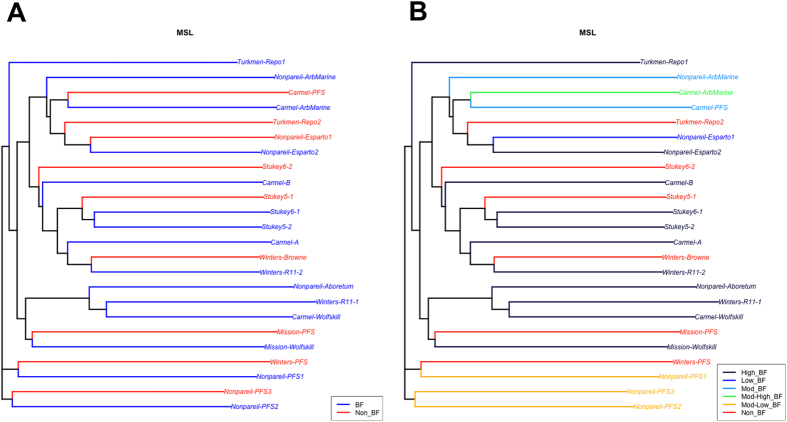
Dendrograms resulted from the Principal Coordinates Analysis performed through MSAP. (**A**) Relationships between *BF* and *NBF* accessions within and between genotypes. (**B**) Distinction among six levels of BF-exhibition in accessions within and between almond cultivars and clone sources based on results from MS-AFLP procedure.

The results from the Principal Coordinates Analysis are shown in the form of dendrograms in Fig. 2, in which a distinction between *BF* and *NBF* accessions per cultivar, and according to the different degrees of BF-exhibition, respectively, is not strongly evident. Attempts to fit BF as a dichotomous response (presence/absence) were done; however, several problems in algorithm convergence and model stability were observed, and for that reason results using the polytomous responses fitted through multinomial models are presented in the next section.

### Bayesian analysis of contingency tables

The analytic assessment through Bayesian inference and MCMC showed that out the five models evaluated with a maximum of twenty-eight parameters, the included double and triple interactions provided the best fit to the data. Such a model provided a posterior probability of 0.955 supported by a Bayesian *p*-value (*p*_*B*_) of 1 for the deviance statistic.

The double and triple interactions showed high evidence (close to probability of 1, Table 3), which immediately suggests that main effects, i.e. the effect of each parameter alone [(DNA-(de)methylation status, clonal age or genotype], becomes of low relevance, since the simple effects, i.e. the responses in BF-exhibition drawn by the interactions, capture and explain greater amount of the information analyzed in the model. The ranking of model terms as determined by their variance contribution to the model total variance is presented in the last column of Table 3. *ChronoAge* is the most important parameter, while the term for the triple interaction; corresponding to the interaction of methylated bands for clones of the cultivar ‘Turkmen’ is the least important.

The statistics for support of results from the hierarchical model selection were significantly supportive for the results. Thus, the *adjusted pseudo-R*^*2*^ of McFadden for this model was 0.8687393. The acceptance rate of the reversible jump proposal was equal to 0%, which means that during the MCMC simulations the chain did not move in the space for all the probable models due to lack of adequacy of the other models. The rate of Metropolis-Hastings proposals was 74.5524%, which means that the majority of values proposed in the MCMC simulations were accepted.

**Figure 3:**
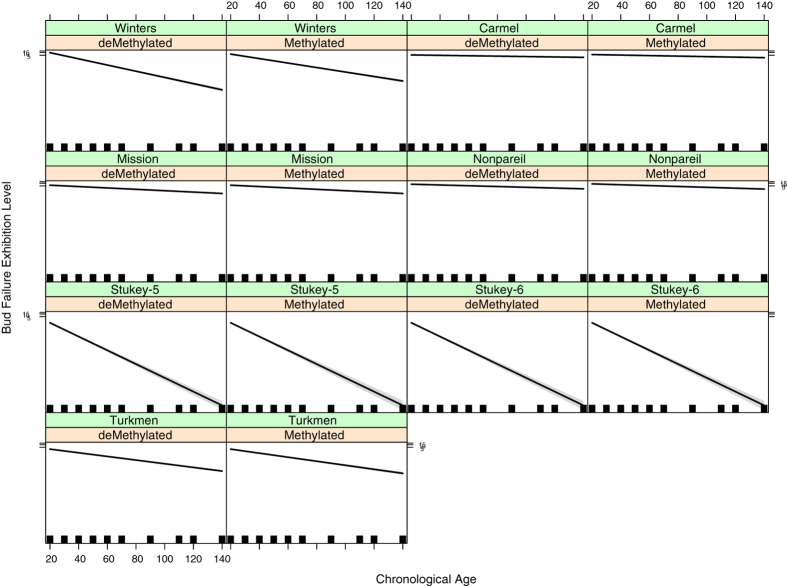
Effect plot showing the interaction of the chronological age (X axis, in years), the DNA-(de)methylation status of the MS-AFLP band (presence absence, 1/0), and the almond cultivar according to the full model. The trend lines in each box pair summarizes the triple-interaction of the chronological age (X axis, in years), the DNA-(de)methylation status of the MS-AFLP band (presence or absence, 1/0), with respect to the almond cultivar evaluated. The slope of the lines indicates the rate of change of DNA-(de)methylation status for each cultivar for each type of band (demethylated and methylated).

In Fig. 3, the effect plot for the visualization of the effect on BF-exhibition by triple interactions is shown. Represented in this figure is each trend line summarizing the triple-interaction action of the chronological age (X axis, in years), the DNA-(de)methylation status of the MS-AFLP band (presence or absence, 1/0), with respect to the almond source clone evaluated. The slope of the lines indicates the rate of change of DNA-(de)methylation status for each source clone for each type of band (demethylated and methylated). It is notable that in all cases the effects were significantly different from zero. In addition, differences in the slopes with respect to DNA-(de)methylation status in intra-cultivar comparison were negligible but significantly distinct in inter-cultivar comparisons, particularly evident for the source clones of ‘Winters’ and Stukey accessions.

Model selection considering only the ‘Nonpareil’ source clones showed that, of the three models evaluated with a maximum of four parameters, the model with the interaction was the most adequate model to fit to the data. This model showed a posterior probability of 0.5541, the second most adequate model considered *deMet01* and *ChronoAge* only, but its posterior probability was of 0.4459. The summary statistics of the log-linear parameters for the most probable model are presented in Table 4. The value for the coefficient of the interaction between chronological age and extent of DNA-(de)methylation was small but positive (0.005994) and with a posterior probability of 0.5541. The *adjusted pseudo-R*^*2*^ of McFadden for this model was 0.74971.

For the hierarchical model selection considering ‘Nonpareil’ accessions, the statistics were significantly supportive for the results. The adequacy of the most probable model is supported by a *p*_*B*_ = 1 for the deviance statistic and an acceptance rate of the reversible jump proposal of 75.5708%. This shows that during the MCMC simulations, the chain moved freely in the values space for all the probable models, the acceptance rate of Metropolis-Hastings proposal was high (92.9962%), and that most of the values proposed in the MCMC simulations were accepted.

**Figure 4:**
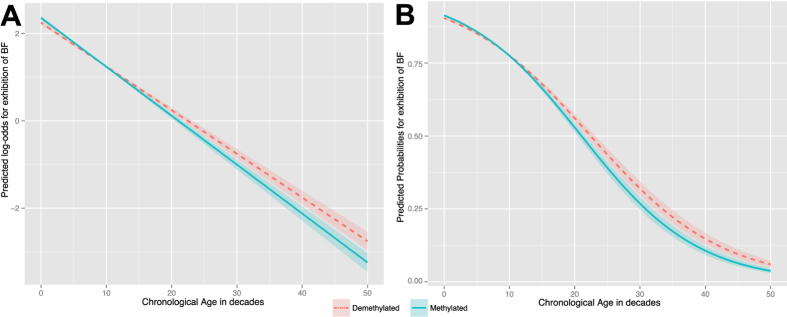
Effects plots considering Chronological age in decades (on X) and DNA-(de)methylations status of MS-AFLP bands for the full model fitting BF in the cultivar ‘Nonpareil’. (**A**) Plot showing the predicted log-odds for the exhibitions of Non-infectious Bud Failure. (**B**) Plot showing the predicted probabilities for the exhibitions of BF.

In Fig. 4, the tendency of the predicted log odds and predicted probabilities for the exhibition of BF in relation to the interaction between chronological age and extent of DNA-(de)methylation for clones of ‘Nonpareil’ is shown. For the case in which DNA is demethylated (band absent = 0), one unit increase in chronological age yields a change in log odds of −0.105934, while where DNA tends to be methylated (band present = 1), one unit increase in chronological age yields a change in log odds of −0.099994. This is translated in terms of odd ratios 0.89948 for one-year increase in chronological age for DNA-demethylation and 0.90489 for DNA-methylation. Significant differences in trends are evident from the 17^th^ decade of chronological age, in which DNA-methylated status exhibits a faster decrease for the log odds of BF with respect to the increase of chronological age (i.e., the relationship is negative). Similarly, the predicted probabilities of BF-exhibition while DNA-methylated status is given, exhibit a faster decrease with respect to the increase of chronological age from the 17^th^ decade, and converging asymptotically with DNA-demethylated status towards a probability of zero by the 70^th^ decade.

## Discussion

The possible association of DNA-(de)methylation with Noninfectious Bud-failure (BF) is significant for applications ranging from practical issues in the management of clonal propagation sources for stable commercial production to strategies for the genetic/genomic improvement and breeding of clonally propagated crops. Research on important but largely neglected topics in clonal crop biology, such as the relationship between ageing and the loss of gene integrity may also be explored.

The results from methylation-sensitive amplified fragment length polymorphism (MS-AFLPs) showed that the technique is informative for almond, which is an obligate outcrosser. A remarkably high level of epigenetic polymorphisms was present. Every primer combinations provided polymorphic bands for an impressive amount of 1129 polymorphic fragments representing the largest amount of polymorphic MS-AFLP bands reported for a plant species. Usually, the number of polymorphic bands has not been more than 30 sites genome-wide in other research reports. Much lower levels of de-methylation were reported between cultivars from two distinct genetic pools in rice^34^, an inbred crop species. Lower levels of de-methylation were also reported within the same cultivar but in different environmental conditions in apple^35^, between landraces and selections in plantains^36^, bananas^37^, and date^38^, between somaclones in grape^39^, and between groups of virus infected and uninfected plants in tomato^40^.

The experimental method followed in the present study requires a very significant effort in scoring and the subsequent identification of consensus bands between the two assays. While automated scoring was attempted, the large number of bands made the task difficult. Therefore, methods allowing high-throughput scoring (such as a capillary based system) would be desirable for future experiments. Despite of the limitations of the MS-AFLP technique (i.e. targeting anonymous genome fragments, lack of predictive model phenotype changes due to DNA methylation changes, validity overtime and scoring based on dominant bands)^41^, MS-AFLP is a useful tool for the exploratory assessment of epigenetic and epigenomic variation in crop species such as almond where high quality genome assemblies and gene annotations are not yet available. Simple but meaningful improvements may be done by increasing the primer combinations with additional non-methylation sensitive enzymes such as *Acc*65I and *Kpn*I with *Mse*I^41^, and even targeting asymmetric DNA methylation by using other enzymes such as *Bg*III, *Bmt*I and *BsaJ*I, which will help to further interrogate methylation patterns in crops.

The patterns of methylation status seen for *BF* and *NBF* accessions suggested departures from independence, but without a clear differentiation based on methylation status only. Thus, *BF* accessions showed a proportion of 0.56 for fragments with some level of methylation, while *NBF* accessions showed a proportion of 0.50 with some level of methylation. HPA-/MSP- sites, which target fully methylated fragments, represented the main source of differentiation between *BF* and *NBF* accessions. Such inconclusive results have also been observed in other attempts to link methylation profile with disease exhibition or the infection from a pathogen, such as tomato yellow leaf curl sardinia virus^40^ or fusarium wilt in chickpea^42^. In the present analysis, this inconsistent pattern is supported through the clustering analysis performed for the levels of BF-exhibition in relation to purely methylation status of genome fragments (Fig. 2).

The finding that bands *e*_22_ and *i*_19_ demonstrated total correspondence with BF-exhibition (presence for *BF* accessions and absence for *NBF* accessions) was particularly interesting and represent targets of future study. The bands were sequenced but the results were inconclusive since no significant alignment was found in a search in GenBank. The strong association of these differentially methylated bands suggests that Methylation-Sensitive Representational Difference Analysis (MS-SRDA)^43^ will provide additional insights on the relationship of methylation changes with BF-exhibition. Another tentative approach would consist of developing PCR bisulfite primers, which would allow high-throughput obtaining of methylation levels in cytosine, in highly-multiplexed manner, and thus to characterize larger sets of samples.

Current techniques, such as diversity array technology (DArT)^44^ and its newest derivation DArTseq which utilizes next generation sequencing platforms^45^ may provide more efficient methylation profiles, while additional genome-wide markers or even methylated sites may be annotated based on a reliable reference genome.

The continued development of improved genome sequencing platform such as the single molecule real time sequencing^46^ should greatly facilitate the sequencing and assembling of high quality heterozygous genomes (such as almond) while improved profiling of methylation status^47^ as well as the application of bisulfite sequencing as recently applied to studies of oil palm^48^. Development of genomic resources in almond will also allow the exploration of the influence of other genetic factors that may be involved in BF and related phenomena; for example, microRNA mediating DNA methylation. Esmaeili *et al*.^49^ provides relevant insights about the role of a specific family of miRNA elements in the response to abiotic stresses in almond. Extensions of this type of research would allow a comprehensive interrogation of the role of biotic stresses such as heat stress in the exhibition of BF, as has been previously suggested^17^,^18^. Surveying miRNAs will also address the role of miRNA in the ageing and longevity of tissue, because, even though Esmaeili *et al*.^49^ tested miR396 and did not obtained association with response to drought, such an miRNA element has been shown to play a role in leaf development and longevity^50^.

The use of Bayesian analysis of contingency tables through hierarchical log-linear models was successful in evaluating the association between methylation status and BF as well as the association between DNA-(de)methylation and clone age. Based on the evidence provided by the dataset generated and analyzed in the present study, DNA-(de)methylation is not independent of the exhibition of noninfectious bud-failure in the almond accessions evaluated. Therefore, there exist an association between DNA-(de)methylation which interacts with other factors such as age and cultivar. However, the association is uneven because of several relevant modulators (e.g. environment and initial cultivar BF-status at the start of propagation) which need to be taken into consideration in future research in order to dissect the effects of the interactions between and among modulators.

The use of a Bayesian approach allows a fuller assessment of the uncertainties related to models and parameter values generated from the contingency tables, as well as the evaluation of every unique combination of interaction in a particular set of models because of the ability to evaluate hierarchical models with distinct parameter spaces (i.e. likelihood functions) simultaneously. It is particularly useful when there is not a previously established model, as is the case of the present study, as it provides a higher level of certainty and robustness in defining the most appropriate model for the dataset provided. The evaluation of the posterior distribution of the model parameters and model indicators using MCMC through the application of algorithms and methods already developed and made available by statisticians, allowed testing of the associations and interactions among DNA-(de)methylation, chronological age to clonal propagation and the exhibition of non-infections bud-failure. When also considering several clone sources, it allowed assessment of three-way interactions, which, for the purpose of expanded experimentation and model development, are crucial sources of information. However, a larger (total and inter genotype) sample size would be beneficial to improve the precision in the estimation of the relevance of the biological factors considered [(DNA-(de)methylation status, clonal age and genotype].

The finding that the saturated model was the most appropriate to fit the data provided is not surprising given the characteristics for the dataset. Almond is a highly heterogeneous crop, which is the result of gametophytic self-incompatibility in its reproduction system and its highly heterozygous nature^51^. Therefore, it might be expected that the epigenetic differentiation between cultivars would also be significant, since methylation profiles have been used to reveal additional hidden variation in homozygous species such as saffron^52^.

It is also relevant that while running MCMC, the lack of adequacy of other models was statistically evident based on the values of acceptance rates for reversible jump proposals (zero), the Metropolis-Hastings proposals (>74%), the Bayesian *p*-value (1) as well as the value of the *adjusted pseudo-R*^*2*^ of McFadden which was approximately 0.86. Consequently, the model captured around 87% of total methylation dependent variation present in the dataset, which directly provided a preliminary hypothesis for critical examination. Using both DNA-(de)methylation and chronological age to analyze BF, the analyses were more effective on a cultivar-by-cultivar (source-by-source, genotype-by-genotype) basis (as supported by the differences in slopes showed in Fig. 3). This indicates that initial BF level as well as BF development varies with cultivar, possibly including cultivar growth and propagation history; however, the effect of environment also needs further investigation.

The availability of extensive research on the exhibition of BF in different cultivars and environments^2^,^17^,^18^,^53^,^54^, has provided some insights about the role of the environment, particularly heat stress during the previous growing season on the rate of BF development and exhibition. This research also demonstrates differences in the BF development and exhibition among source clones within cultivars^55^. For the commercially dominant cultivar ‘Nonpareil’, which constitutes 40% of the acreage^21^, the extent of genome-wide DNA-(de)methylation and its interaction with chronological age appears to play a critical role in the degree of BF-exhibition. So far, chronological age from initial clonal propagation seems to be a relevant factor for the development of BF. This is consistent with a gradual accumulation of both methylation status and BF susceptibility with time, since evaluated accessions of ‘Nonpareil’ date back to near its initial discovery and introduction in California in 1886 [ref. 56]. Previous research has shown that almond cultivars differ in their BF susceptibility at the time of their initial selection (usually from seedling populations)^15^. For example, ‘Carmel’ is a more recent introduction, having been first commercially propagated in the 1960s, though all propagation clonal sources currently used by commercial nurseries, including the original seedling tree, show higher levels of BF than much older Nonpareil clonal sources do. Because both initial BF susceptibility as well as propagation history will differ among cultivars, effective predictors of current BF susceptibility performance will need to be developed on a cultivar-by-cultivar basis.

The possible use of changes in DNA-(de)methylation patterns as a predictor of plant clonal age provides novel research opportunities. One example is the pursuit for the “ontogenetic molecular clock”, given that plant ageing occurs chronologically and ontogenetically in a concurrent but in an opposite way^57^, due to the indeterminacy and totipotency of meristems^8^. It remains a challenge to define the ontogenetical, or at least physiological, age of plant tissues of clonal propagated perennial tree crops, which we consider would be appropriate to approach disorders related with ageing such as BF in almond. In other crops such as cereals and some forest trees, the phylotaxis summarized in the concept of plastochron index^58^ was developed as an alternative to measure age of plants; however, that approach, as well the ontogenetic index^59^, only considers plants derived from sexual propagation, and for which good approximations to the number of leaves can be done. The tracking of changes in patterns and amount of DNA-(de)methylation in successive clonal propagation sources and productive clones may aid to understand ageing in clonally propagated perennial tree crops. The accurate age measurement is relevant because in trees and other long-lived plants, epicormic meristems at the base of the plant can maintain their more juvenile state. Indeed, most current commercial clones or sources of propagation material for the cultivar ‘Nonpareil’ originated as basal epicormic shoots for individual trees approaching 10 decades (~100 years) in age, and so from plantings made shortly after the introduction of the cultivar. Although these trees may be exhibiting BF in its uppermost sections, trees propagated from foundation stock developed in the basal epicormic shoots have consistently shown BF free development over many decades, as well as many propagation cycles^60^.

In conclusion, these research results provide a strategy for the study of Noninfectious Bud Failure (BF) based on methylation changes across the almond genome, which shows promise for both applied and basic future research. These findings also provide a model for understanding plant ageing in perennial clonal crops, and may provide the basis for effective selection against epigenetic-like disorders such as BF in almond as well as ‘Rusty-Blotch’ and ‘False Shot-Hole’ disease in plums, ‘Russet-Scab’ in prune, ‘Gumboil’ in apricot, and ‘Crinkle-Leaf’ disease in cherry and plum. Findings also suggest previously unexploited opportunities in the selection for similar epigenetic-like factors with positive contributions to plant performance and final yield.

## Additional Information

**How to cite this article:** Fresnedo-Ramírez, J. *et al*. Genome-wide DNA-(de)methylation is associated with Noninfectious Bud-failure exhibition in Almond (*Prunus dulcis* [Mill.] D.A.Webb). *Sci. Rep.* **7**, 42686; doi: 10.1038/srep42686 (2017).

**Publisher's note:** Springer Nature remains neutral with regard to jurisdictional claims in published maps and institutional affiliations.

## Supplementary Material

Supplementary File

Supplementary Data

## Figures and Tables

**Table 1:** Accession ID, cultivar, location, BF exhibitions and chronological age from initial clonal propagation (clone source age) for accession analyzed in the present study.

Accession ID	Cultivar or Genotype	Location and GPS coordinates	BF exhibition	Chronological Age (decades)
Carmel-ArbMarine	Carmel	Nickels Soils Lab., Arbuckle, CA *N38° 57.504′ W122° 04.004′*	Medium to High	2
Carmel-PFS	Carmel	Plant Foundation Services, Davis, CA *N38° 31.642′ W121° 46.380′*	Medium	7
Carmel-Wolfskill	Carmel	Wolfskill Exptl. Orchard, Winters, CA *N38° 30.343′ W121° 58.567′*	High	3
Mission-PFS	Mission	Plant Foundation Services, Davis, CA *N38° 31.642′ W121° 46.380′*	No	12
Mission-Wolfskill	Mission	Wolfskill Exptl. Orchard, Winters, CA *N38° 30.343′ W121° 58.567′*	High	2
Nonpareil-ArbMarine	Nonpareil	Nickels Soils Lab., Arbuckle, CA *N38° 57.504′ W122° 04.004′*	Low to Medium	9
Nonpareil-Arboretum	Nonpareil	Nickels Soils Lab., Arbuckle, CA *N38° 57.504′ W122° 04.004′*	High	5
Nonpareil-Esparto1	Nonpareil	Esparto, CA *N38° 41.631′ W122° 01.344′*	Low	11
Nonpareil-Esparto2	Nonpareil	Esparto, CA *N38° 41.631′ W122° 01.344′*	High	6
Nonpareil-PFS1	Nonpareil	Plant Foundation Services, Davis, CA *N38° 31.642′ W121° 46.380′*	Low to Medium	14
Nonpareil-PFS2	Nonpareil	Plant Foundation Services, Davis CA *N38° 31.642′ W121° 46.380′*	Low to Medium	12
Nonpareil-PFS3	Nonpareil	Plant Foundation Services, Davis, CA *N38° 31.642′ W121° 46.380′*	Low to Medium	12
Stukey-5-1	Stukey-5	Wolfskill Exptl. Orchard, Winters, CA *N38° 30.343′ W121° 58.567′*	Low	2
Stukey-5-2	Stukey-5	Wolfskill Exptl. Orchard, Winters, CA *N38° 30.343′ W121° 58.567′*	High	1
Stukey-6-1	Stukey-6	Wolfskill Exptl. Orchard, Winters, CA *N38° 30.343′ W121° 58.567′*	High	1
Stukey-6-2	Stukey-6	Wolfskill Exptl. Orchard, Winters, CA *N38° 30.343′ W121° 58.567′*	Low	2
Turkmen-Repo1	Turkmen	National Clonal Germplasm, Winters, CA *N38° 29.842′ W121° 58.611′*	High	1
Turkmen-Repo2	Turkmen	National Clonal Germplasm, Winters, CA *N38° 29.842′ W121° 58.611′*	Low	2
Winters-Browne	Winters	Sanger, CA *N36° 42.863′ W119° 32.061′*	No	4
Winters-PFS	Winters	Plant Foundation Services, Davis, CA	No	5
Winters-R11-1	Winters	Sanger, CA *N36° 42.863′ W119° 32.061′*	High	3
Winters-R11-2	Winters	Sanger, CA *N36° 42.863′ W119° 32.061′*	High	2

**Table 2:** Methylation levels per type of primer combination for each type of methylation status of the loci, the proportion of the banding is showed conditional to presence or absence of BF symptoms.

Type of primer combination	Methylation status of the loci	Proportion in *BF* accessions	Proportion in *NBF* accessions
HPA+/MSP+	Demethylated	0.439083	0.500477
HPA+/MSP−	Hemimethylated	0.085261	0.093192
HPA−/MSP+	Internal cytosine methylation	0.095542	0.111845
HPA−/MSP−	Full methylation	0.380113	0.294485

**Table 3:** Statistics calculated from the posterior distribution of the parameters included in the most probable model for the 22 almond accessions.

Parameter	Posterior Probability	Posterior Mean	Posterior Variance	Lower Limit	Upper Limit	Ranking VarImp
Intercept	1.000	2.721365	1.101e-04	2.700337	2.741896	NA
Factor(deMet01)1	1.000	0.035703	1.170e-04	0.014644	0.058054	14
ChronoAge	1.000	−0.731076	4.816e-05	−0.745108	−0.717645	1
Factor(GenotypeN)2	1.000	0.741199	9.978e-04	0.683076	0.803948	4
Factor(GenotypeN)3	1.000	−0.884685	2.887e-04	−0.920034	−0.854581	7
Factor(GenotypeN)4	1.000	−0.572292	2.954e-04	−0.607085	−0.540216	8
Factor(GenotypeN)5	1.000	−0.421255	3.136e-04	−0459597	−0.389653	24
Factor(GenotypeN)6	1.000	0.858615	1.145e-03	0.795386	0.928581	25
Factor(GenotypeN)7	1.000	0.859652	1.183e-03	0.793455	0.926133	6
Factor(deMet01)1:ChronoAge	1.000	−0.010859	5.082e-05	−0–025151	0.002451	19
Factor(deMet01)1:factor(GenotypeN)2	1.000	0.377405	9.582e-04	0.318386	0.438253	12
Factor(deMet01)1:factor(GenotypeN)3	1.000	−0.100029	3.040e-04	−0.134473	−0.066831	16
Factor(deMet01)1:factor(GenotypeN)4	1.000	−0.036256	2.897e-04	−0.067587	−0.001094	13
Factor(deMet01)1:factor(GenotypeN)5	1.000	−0.091411	3.133e-04	−0.126620	−0.058245	22
Factor(deMet01)1:factor(GenotypeN)6	1.000	−0.034131	1.221e-03	−0.097879	0.037375	23
Factor(deMet01)1:factor(GenotypeN)7	1.000	−0.033510	1.188e-03	−0.099132	0.034752	15
ChronoAge:factor(GenotypeN)2	1.000	−0.037135	1.592e-04	0.010168	0.059298	2
ChronoAge:factor(GenotypeN)3	1.000	0.671377	1.592e-05	0.656178	0.686165	5
ChronoAge:factor(GenotypeN)4	1.000	0.552007	5.619e-05	0.538380	0.567475	3
ChronoAge:factor(GenotypeN)5	1.000	0.625152	5.064e-05	0.612284	0.639953	9
ChronoAge:factor(GenotypeN)6	1.000	−1.057749	7.848e-04	−1.110633	−1.002450	10
ChronoAge:factor(GenotypeN)7	1.000	−1.058923	7.621e-04	−1.112500	−1.005495	11
Factor(demet01)1:ChronoAge:factor(GenotypeN)2	1.000	−0.100419	1.526e-04	−0.122971	−0.075562	18
Factor(demet01)1:ChronoAge:factor(GenotypeN)3	1.000	0.018288	6.113e-05	0.002320	0.032875	20
Factor(demet01)1:ChronoAge:factor(GenotypeN)4	1.000	0.010936	5.863e-05	−0.003976	0.025603	17
Factor(demet01)1:ChronoAge:factor(GenotypeN)5	1.000	0.016861	5.261e-05	0.003079	0.031003	27
Factor(demet01)1:ChronoAge:factor(GenotypeN)6	1.000	0.009808	8.472e-04	−0.045120	0.068286	26
Factor(demet01)1:ChronoAge:factor(GenotypeN)7	1.000	0.009048	7.905e-04	−0.051850	0.059506	27

The values for the lower and upper limits correspond to the 95% highest posterior density intervals, respectively. Ranking VarImp refers to the ranking of variance contribution to the model: lower values being top contributors and higher values being least contributors.

**Table 4:** Statistics calculated form the posterior distribution of the parameters included in the most probable model for the exhibition of BF in ‘Nonpareil’ accessions.

Parameter	Posterior Probability	Posterior Mean	Posterior Variance	Lower Limit	Upper Limit
Intercept	1.0000	2.300100	3.126e-04	2.265517	2.333353
Factor(deMet01)	1.0000	−0.055689	8.872e-04	−0.089676	−0.023565
ChronoAge	1.0000	−0.105934	3.575e-06	−0.109714	−0.102453
Factor(deMet01)1:ChronoAge	0.5541	0.005994	3.295e-06	0.002334	0.009363

The values for the lower and upper limits correspond to the 95% highest posterior density intervals, respectively.

## References

[b1] Fenton C. A. L., Kuniyuki A. H. & Kester D. E. Search for a viroid etiology for noninfectious bud failure in almond. HortScience 23, 1050–1053 (1988).

[b2] Kester D., Shackel K., Micke W., Cunningham M. & Gradziel T. The Noninfectious Bud-failure problem in almonds: An interaction of unique biological, adaptive and cultural conditions. HortScience 38, 726–726 (2003).

[b3] Ogawa J. M. & English H. Diseases of Temperate Zone Tree Fruit and Nut Crops. (Publications University of California Division of Agriculture and Natural Resources, 1991).

[b4] Campisi J. & Vijg J. Does Damage to DNA and Other Macromolecules Play a Role in Aging? If So, How? J Gerontol A Biol Sci Med Sci 64, 175–178 (2009). 19228786 10.1093/gerona/gln065 PMC2655027

[b5] D’Aquila P., Rose G., Bellizzi D. & Passarino G. Epigenetics and aging. Maturitas 74, 130–136 (2013). 23245587 10.1016/j.maturitas.2012.11.005

[b6] Fraga M. F., Rodriguez R. & Canal M. J. Genomic DNA methylation-demethylation during aging and reinvigoration of *Pinus radiata*. Tree Physiol 22, 813–816 (2002). 12184986 10.1093/treephys/22.11.813

[b7] Garinis G. A., van der Horst G. T. J., Vijg J. & Hoeijmakers J. H. J. DNA damage and ageing: new-age ideas for an age-old problem. Nat Cell Biol 10, 1241–1247 (2008). 18978832 10.1038/ncb1108-1241 PMC4351702

[b8] Munne-Bosch S. Do perennials really senesce? Trends Plant Sci 13, 216–220 (2008). 18328774 10.1016/j.tplants.2008.02.002

[b9] Thomas H. Aging in the plant and animal kingdoms – the role of cell death. Rev Clin Gerontol 4, 5–20 (1994).

[b10] Thomas H. In Model Systems in Aging Vol. 3 Topics in Current Genetics Ch. 6, 145–171 (Springer: Berlin Heidelberg, 2004).

[b11] Finnegan E. J., Genger R. K., Kovac K., Peacock W. J. & Dennis E. S. DNA methylation and the promotion of flowering by vernalization. Proc. Natl. Acad. Sci. USA 95, 5824–5829 (1998). 9576969 10.1073/pnas.95.10.5824 PMC20464

[b12] Oliver S. N., Finnegan E. J., Dennis E. S., Peacock W. J. & Trevaskis B. Vernalization-induced flowering in cereals is associated with changes in histone methylation at the VERNALIZATION1 gene. Proc. Natl. Acad. Sci. USA 106, 8386–8391 (2009). 19416817 10.1073/pnas.0903566106 PMC2677093

[b13] Trap-Gentil M. V. . Time course and amplitude of DNA methylation in the shoot apical meristem are critical points for bolting induction in sugar beet and bolting tolerance between genotypes. J. Exp. Bot. 62, 2585–2597 (2011). 21227931 10.1093/jxb/erq433

[b14] Kester D. E. Solving the problem of noninfectious bud-failure in California almond orchards. Acta Hortic 373, 35–39 (1994).

[b15] Kester D. E., Shackel K. A., Micke W. C., Gradziel T. M. & Viveros M. Variability in potential and expression of noninfectious bud-failure among nursery propagules of ‘Carmel’ almond. Acta Hortic 470, 268–272 (1998).

[b16] Kester D. E. Comparative inheritance of Noninfectious bud-failure (BF) in almond × almond and almond × peach progenies. HortScience 13, 372–372 (1978).

[b17] Kester D. E. & Asay R. N. Variability in Noninfectious bud-failure of nonpareil almond. 1. Location and environment. J Am Soc Hortic Sci 103, 377–382 (1978).

[b18] Kester D. E. & Asay R. N. Variability in Noninfectious bud-failure of nonpareil almond. 2. Propagation source. J Am Soc Hortic Sci 103, 429–432 (1978).

[b19] Gradziel T. M. In Breeding Plantation Tree Crops: Temperate Species Breeding Plantation Tree Crops (eds Priyadarshan M. & Jain S. M.) 1–31 (Springer: New York, 2009).

[b20] Sathe S. K., Teuber S. S., Gradziel T. M. & Roux K. H. Electrophoretic and immunological analyses of almond (*Prunus dulcis* L.) genotypes and hybrids. J Agr Food Chem 49, 2043–2052 (2001). 11308365 10.1021/jf001303f

[b21] Almond Board of California. Almond Almanac for 2014: http://www.almonds.com/sites/default/files/content/attachments/2014_almanac_final.pdf (2014).

[b22] Martínez-Gómez P. & Gradziel T. M. Sexual polyembryony in almond. Sex Plant Reprod 16, 135–139 (2003).

[b23] Doyle J. J. & Doyle J. L. Isolation of plant DNA from fresh tissue. Focus 12, 13–15 (1990).

[b24] Keb-Llanes M., González G., Chi-Manzanero B. & Infante D. A rapid and simple method for small-scale DNA extraction in *Agavaceae* and other tropical plants. Plant Mol Biol Rep 20, 299–299 (2002).

[b25] Perez-Figueroa A. msap: a tool for the statistical analysis of methylation-sensitive amplified polymorphism data. Mol Ecol Resour 13, 522–527 (2013). 23311622 10.1111/1755-0998.12064

[b26] R: A Language and Environment for Statistical Computing v. 3.0.1 (Development Core Team R, Viena, Austria, 2013).

[b27] Meyer D., Zeileis A. & Hornik K. The Strucplot Framework: Visualizing Multi-way Contingency Tables with vcd. J Stat Softw 17, 1–48 (2006).

[b28] Zeileis A., Meyer D. & Hornik K. Residual-Based Shadings for Visualizing (Conditional) Independence. J Comput Graph Stat 16, 507–525 (2007).

[b29] Overstall A. M. & King R. Conting: An R Package for Bayesian Analysis of Complete and Incomplete Contingency Tables. J Stat Softw 58, 1–27 (2014).

[b30] Link W. A. & Eaton M. J. On thinning of chains in MCMC. Methods Ecol Evol 3, 112–115 (2012).

[b31] Sabanés-Bové D. & Held L. Hyper-g Priors for Generalized Linear Models. Bayesian Anal 6, 387–410 (2011).

[b32] McCullagh P. & Nelder J. A. Generalized linear models. 2nd edn (Chapman & Hall/CRC, 1998).

[b33] McFadden D. In Behavioural travel modelling (eds Hensher D. A. & Stopher P. R.) 279–318 (Croom Helm, 1979).

[b34] Ashikawa I. Surveying CpG methylation at 5′-CCGG in the genomes of rice cultivars. Plant Mol Biol 45, 31–39 (2001). 11247604 10.1023/a:1006457321781

[b35] Li X., Xu M. & Korban S. S. DNA methylation profiles differ between field- and *in vitro*-grown leaves of apple. J Plant Physiol 159, 1229–1234 (2002).

[b36] Noyer J. L., Causse S., Tomekpe K., Bouet A. & Baurens F. C. A new image of plantain diversity assessed by SSR, AFLP and MSAP markers. Genetica 124, 61–69 (2005). 16011003 10.1007/s10709-004-7319-z

[b37] Gimenez C., Palacios G. & Colmenares A. *Musa* methylated DNA sequences associated with tolerance to *Mycosphaerella fijiensis* toxins. Plant Mol Biol Rep 24, 33–43 (2006).

[b38] Fang J. G. & Chao C. T. Methylation-sensitive amplification polymorphism in date palms (*Phoenix dactylifera* L.) and their off-shoots. Plant Biol 9, 526–533 (2007). 17642034 10.1055/s-2007-964934

[b39] Schellenbaum P., Mohler V., Wenzel G. & Walter B. Variation in DNA methylation patterns of grapevine somaclones (*Vitis vinifera* L.). BMC Plant Biol 8, 78 (2008). 18627604 10.1186/1471-2229-8-78 PMC2491626

[b40] Mason G. . Potentiality of Methylation-sensitive Amplification Polymorphism (MSAP) in Identifying Genes Involved in Tomato Response to Tomato Yellow Leaf Curl Sardinia Virus. Plant Mol Biol Rep 26, 156–173 (2008).

[b41] Schrey A. W. . Ecological Epigenetics: Beyond MS-AFLP. Integr Comp Biol 53, 340–350 (2013). 23583961 10.1093/icb/ict012

[b42] Mohammadi P., Bahramnejad B., Badakhshan H. & Kanouni H. DNA methylation changes in fusarium wilt resistant and sensitive chickpea genotypes (*Cicer arietinum* L.). Physiol Mol Plant Path 91, 72–80 (2015).

[b43] Ushijima T. & Yamashita S. In DNA Methylation: Methods and Protocols (ed Tost Jörg) 117–130 (Humana Press, 2009).

[b44] Jaccoud D., Peng K., Feinstein D. & Kilian A. Diversity arrays: a solid state technology for sequence information independent genotyping. Nucleic Acids Res 29, E25 (2001). 11160945 10.1093/nar/29.4.e25 PMC29632

[b45] Kilian A. . In Data Production and Analysis in Population Genomics Vol. 888 Methods in Molecular Biology (eds Pompanon François & Bonin Aurélie) Ch. 5, 67–89 (Humana Press, 2012).

[b46] Eid J. . Real-time DNA sequencing from single polymerase molecules. Science 323, 133–138 (2009). 19023044 10.1126/science.1162986

[b47] Krebes J. . The complex methylome of the human gastric pathogen *Helicobacter pylori*. Nucleic Acids Res 42, 2415–2432 (2014). 24302578 10.1093/nar/gkt1201 PMC3936762

[b48] Ong-Abdullah M. . Loss of Karma transposon methylation underlies the mantled somaclonal variant of oil palm. Nature 525, 533–537 (2015). 26352475 10.1038/nature15365 PMC4857894

[b49] Esmaeili F. . In silico search and biological validation of microRNAs related to drought response in peach and almond. Funct. Integr. Genomics. 10.1007/s10142-016-0488-x (2016). 27068847

[b50] Debernardi J. M. . Post-transcriptional control of GRF transcription factors by microRNA miR396 and GIF co-activator affects leaf size and longevity. Plant J 79, 413–426 (2014). 24888433 10.1111/tpj.12567

[b51] Rahemi A. . Genetic diversity of some wild almonds and related *Prunus* species revealed by SSR and EST-SSR molecular markers. Plant Syst Evol 298, 173–192 (2012).

[b52] Busconi M. . AFLP and MS-AFLP Analysis of the Variation within Saffron Crocus (*Crocus sativus* L.) Germplasm. PLoS ONE 10, e0123434 (2015). 25885113 10.1371/journal.pone.0123434 PMC4401542

[b53] Kester D. E., Shackel K. A., Gradziel T. M., Micke W. C. & Viveros M. Final results of propagation experiments to show distribution of noninfectious bud-failure in ‘Carmel’ almond. HortScience 33, 526–526 (1998).

[b54] Kester D. E., Shackel K. A., Micke W. C., Viveros M. & Gradziel T. M. Noninfectious bud failure in ‘Carmel’ almond. I. Pattern of development in vegetative progeny trees. J Am Soc Hortic Sci 129, 244–249 (2004).

[b55] Wilson E. E. & Schein R. D. The nature and development of noninfectious bud failure of almonds. Hilgardia 24, 519–542 (1956).

[b56] Wickson E. J. The California fruits and how to grow them. 1st edn (Dewey & Co. Pacific Rural Press, 1889).

[b57] Poethig R. S. Phase change and the regulation of developmental timing in plants. Science 301, 334–336 (2003). 12869752 10.1126/science.1085328

[b58] Meicenheimer R. D. The Plastochron Index: Still Useful after Nearly Six Decades. Am J Bot 101, 1821–1835 (2014). 25366849 10.3732/ajb.1400305

[b59] Climent J., Dantas A. K., Alia R. & Majada J. Clonal variation for shoot ontogenetic heteroblasty in maritime pine (*Pinus pinaster* Ait.). Trees-Struct Funct 27, 1813–1819 (2013).

[b60] Kester D. E., Micke W. C., Viveros M. & Fenton C. A. L. Application of a noninfectous budfailure model for source selection in Nonpareil almond. HortScience 22, 1082–1082 (1987).

