# Supplementary material for: Genome-wide DNA-(de)methylation is associated with Noninfectious Bud-failure exhibition in Almond (Prunus dulcis [Mill.] D.A.Webb)
Source: Sci Rep. 2017 Feb 16;7:42686. doi: 10.1038/srep42686 (PMC5311954; doi:10.1038/srep42686)
Supplement: Supplementary File [file srep42686-s1.pdf]

Genome-wide DNA-(de)methylation is associated with Noninfectious Bud-failure exhibition in Almond [*Prunus dulcis* (Mill.) D.A.Webb]

Authors: **Jonathan Fresnedo-Ramírez[1§¶], Helen M. Chan[1§], Dan E. Parfitt[1], Carlos H. Crisosto[1], Thomas M. Gradziel[1*]**

[1] Department of Plant Sciences, University of California, Davis, CA 95616, USA

[§] Both authors contributed equally to the present study

[*] Corresponding author

[¶] Current address: Department of Horticulture and Crop Science, The Ohio State University/OARDC, Wooster, OH 44691, USA

Email addresses:

JFR: fresnedoramirez.1@osu.edu

HMC: hmchan@ucdavis.edu

DEP: deparfitt@ucdavis.edu

CHC: chcrisosto@ucdavis.edu

TMG: tmgradziel@ucdavis.edu

Supplementary File.pdf

MS-AFLP protocol ....**Page 2**
Table S1: *Primer sequences for MS-AFLP*.......................................**Page 3**
Table S2: *Adaptor sequences for MS-AFLP*.....................................**Page 3**
Table S3: *Primer set and combinations using for MS-AFLP*..................**Page 4**
LI-COR Images of MS-AFLP bands...**Page 5**
Figure S1: *Mosaic plot for departure from independence between BF-exhibition and DNA-(de)methylation*..**Page 24**
Table S4: *Kendall τ correlation coefficients to assess the concordance in patterns of DNA-(de)methylation between almond genotypes*.............................**Page 25**
Table S5: *Kendall τ correlation coefficients for the pairwise concordance in patterns of DNA-(de)methylation among the clonal sources*...................................**Page 26**

MS-AFLP protocol

AFLP analysis was performed as described by Sorkheh et al.[1] to confirm the identity of the clones for each almond cultivar (data not shown). The MS-AFLP analysis was preformed based on the procedure developed by Reyna Lopez et al.[2] for fungi, which is a modification of the standard AFLP protocol developed by Vos et al.[3] by incorporating the use of methylation-sensitive restriction enzymes. In the present, the methods of Portis et al.[4] for peppers and Hughes-Murphree [5] for peaches were used as reference. Thus, 300 ng of genomic DNA were double digested with *Eco*RI with *Hpa*II or *Msp*I restriction enzyme (New England BioLabs, Beverly, MA, USA). The *Eco*RI and *Hpa*II/*Msp*I oligos (Table S1) were annealed at 95 °C for 5 minutes, then transferred to ice for 10 minutes. The final concentrations for the *Eco*RI adaptor and *Hpa*II/*Msp*I adaptor were 5 µM and 50 µM, respectively. One microliter of each adaptor was ligated to 300 ng of *Eco*RI and *Hpa*II restriction digested DNA fragments via T4 DNA ligase (New England BioLabs, Beverly, MA, USA) according to the manufacturer's instructions, with an additional incubation period of 5 minutes at 72 ºC. This process was repeated with a second set of *Eco*RI and *Msp*I restriction digested DNA fragments. The ligation mixture was diluted 10-fold and amplified with complimentary *Eco*RI+A and *Hpa*II/*Msp*I+T primers according to Portis et al.[4] and Hughes-Murphree [5], with modified PCR conditions: 94 °C for 30 s, 56 °C for 60 s, 72 °C for 60 s for 30 cycles, 72 °C for 5 min, and held at 4 °C. The resulting PCR products were diluted 30-fold then re-amplified with primers *Eco*RI+ANN and *Hpa*II/MspI+TNN, with the use of the following modified touchdown PCR conditions: 94 °C for 2 min; 94 °C for 30 s; 65 °C for 30 s, reduced by 0.7 °C per cycle for 12 cycles; 72 °C for 1 min; 94 °C for 30 s; 56 °C for 30 s; 72 °C for 1 min, repeating the cycle 29 times; 72 °C for 2 min; and maintained at 4 °C afterwards. The pre-selective PCR master mix contained 1X Taq buffer,

0.2 U Taq polymerase, 2 mM MgCl2, 0.15 mM dNTPs, 0.2 µM *Hpa*II/*Msp*I+T primer, and 0.2 µM *Eco*RI+A primer. The selective PCR master mix consisted of 1X Taq buffer, 0.5 U Taq polymerase, 2 mM $MgCl_2$, 0.15 mM dNTPs, 0.2 µM *Hpa*II/*Msp*I+TNN primer, 0.04 µM EcroI+ANN primer.

Primers were ordered from MWG/Operon (Louisville, KY, USA) and both *Eco*RI+ACG and *Eco*RI+AAC were 5' labeled with fluorophore [AMINOC6+DY682]. Both *Eco*RI+ACT and *Eco*RI+AGT was 5' labeled with fluorophore [AMINOC6+DY782]. The *Eco*RI and *Hpa*II/*Msp*I oligo sequences used for adaptor construction (Table S2) were obtained from Portis et al.[4]. All resulting MS-AFLP product bands were visualized on a LI-COR DNA analyzer (LI-COR, Lincoln, NE, USA) according to the manufacturer's instructions.

Table S1: Primer sequences for MS-AFLP.

***Eco*RI=E=5'-GACTGCGTACCAATTC**	***Hpa*II/*Msp*I=HM=5'-ATCATGAGTCCTGCTCGG**
**[AMINOC6+DY682]E-ACG**	HM-TAA
**[AMINOC6+DY782]E-ACT**	HM-TCC
**[AMINOC6+DY682] E-AAC**	HM-TTC
**[AMINOC6+DY782]E-AGT**	

Table S2: Adaptor sequences.

	**Forward**	**Reverse**
***Eco*RI**	5'-CTCGTAGACTGCGTACC-3'	5' –AATTGGTACGCAGCAGTCTAC-3'
***Hpa*II/*Msp*I**	5'-GATCATGAGTCCTGCT-3'	5' –CGAGCAGGACTCATGA-3'

Table S3: Primer sets and their respective combinations used in the present study.

Primer sets in order they were scored	Primer sequence E	Primer sequence HM
a	E-ACG	HM-TTC
b	E-AAC	HM-TTC
c	E-AAC	HM-TCC
d	E-ACT	HM-TCC
e	E-AGT	HM-TAA
f	E-ACC	HM-TAA
g	E-ACT	HM-TTC
h	E-ACT	HM-TAA
i	E-ACG	HM-TAA

REFERENCES

1 Sorkheh, K. *et al.* Amplified fragment length polymorphism as a tool for molecular characterization of almond germplasm: genetic diversity among cultivated genotypes and related wild species of almond, and its relationships with agronomic traits. *Euphytica* **156**, 327-344 (2007).

2 Reyna Lopez, G. E., Simpson, J. & Ruiz Herrera, J. Differences in DNA methylation patterns are detectable during the dimorphic transition of fungi by amplification of restriction polymorphisms. *Mol Gen Genet* **253**, 703-710 (1997).

3 Vos, P. *et al.* AFLP - a New Technique for DNA-Fingerprinting. *Nucleic Acids Res* **23**, 4407-4414 (1995).

4 Portis, E., Acquadro, A., Comino, C. & Lanteri, S. Analysis of DNA methylation during germination of pepper (*Capsicum annuum* L.) seeds using methylation-sensitive amplification polymorphism (MSAP). *Plant Sci* **166**, 169-178 (2004).

5 Hughes-Murphree, S. *Analysis of DNA Methylation Differerences Between High and Low Chill Peach (Prunus persica [L. Batsch])* Master of Science (MS) thesis, Clemson University, (2011).

Key for Lane ID location and DNA source order (genotype) on the LI-COR images used to collect the MS-AFLP banding data in the present study. DNA sources indicated with a * where not part of the present study. Primer sets used, as showed in Supplemental File 1 Table S3 are showed at the bottom of the images.

Lane ID	DNA source	Cutter enzyme
1H	Carmel_Wolfskill	HPA
1M	Carmel_Wolfskill	MSP
2H	Carmel_ArbMarine	HPA
2M	Carmel_ArbMarine	MSP
3H	Carmel_PFS	HPA
3M	Carmel_PFS	MSP
4H	Drake_Base*	HPA
4M	Drake_Base*	MSP
5H	Drake_Middle*	HPA
5M	Drake_Middle*	MSP
6H	Drake_Top*	HPA
6M	Drake_Top*	MSP
7H	Mission_Wolfskill	HPA
7M	Mission_Wolfskill	MSP
8H	Mission_PFS	HPA
8M	Mission_PFS	MSP
9H	Nonpareil_Esparto1	HPA
9M	Nonpareil_Esparto1	MSP
10H	Nonpareil_Arboretum	HPA
10M	Nonpareil_Arboretum	MSP
11H	Nonpareil_Esparto2	HPA
11M	Nonpareil_Esparto2	MSP
12H	Nonpareil_ArbMarine	HPA
12M	Nonpareil_ArbMarine	MSP
13H	Nonpareil_PFS1	HPA
13M	Nonpareil_PFS1	MSP
14H	Nonpareil_PFS2	HPA
14M	Nonpareil_PFS2	MSP
15H	Nonpareil_PFS3	HPA
15M	Nonpareil_PFS3	MSP
16H	Turkmen_Repo1	HPA
16M	Turkmen_Repo1	MSP
17H	Turkmen_Repo2	HPA
17M	Turkmen_Repo2	MSP
18H	Peerless_Base*	HPA
18M	Peerless_Base*	MSP
**Continues:**		
19H	Peerless_Middle*	HPA
19M	Peerless_Middle*	MSP
20H	Peerless_Top*	HPA
20M	Peerless_Top*	MSP
21H	Primal_161*	HPA
21M	Primal_161*	MSP
22H	Primal_164*	HPA
22M	Primal_164*	MSP
23H	Primal_192*	HPA
23M	Primal_192*	MSP
24H	Primal_209*	HPA
24M	Primal_209*	MSP
25H	Stukey5_1	HPA
25M	Stukey5_1	MSP
26H	Stukey5_2	HPA
26M	Stukey5_2	MSP
27H	Stukey6_1	HPA
27M	Stukey6_1	MSP
28H	Stukey6_2	HPA
28M	Stukey6_2	MSP
29H	Winters_PFS	HPA
29M	Winters_PFS	MSP
29A H	Monterey	HPA
29A M	Monterey	MSP
30A H	Monterey_Pseudo	HPA
30A M	Monterey_Pseudo	MSP
31H	Winters_R11_2	HPA
31M	Winters_R11_2	MSP
32H	Winters_R11_1	HPA
32M	Winters_R11_1	MSP
33H	Winters_Browne	HPA
33M	Winters_Browne	MSP
34H	Lost_ID_A	HPA
34M	Lost_ID_A	MSP
35H	Lost_ID_B	HPA
35M	Lost_ID_B	MSP

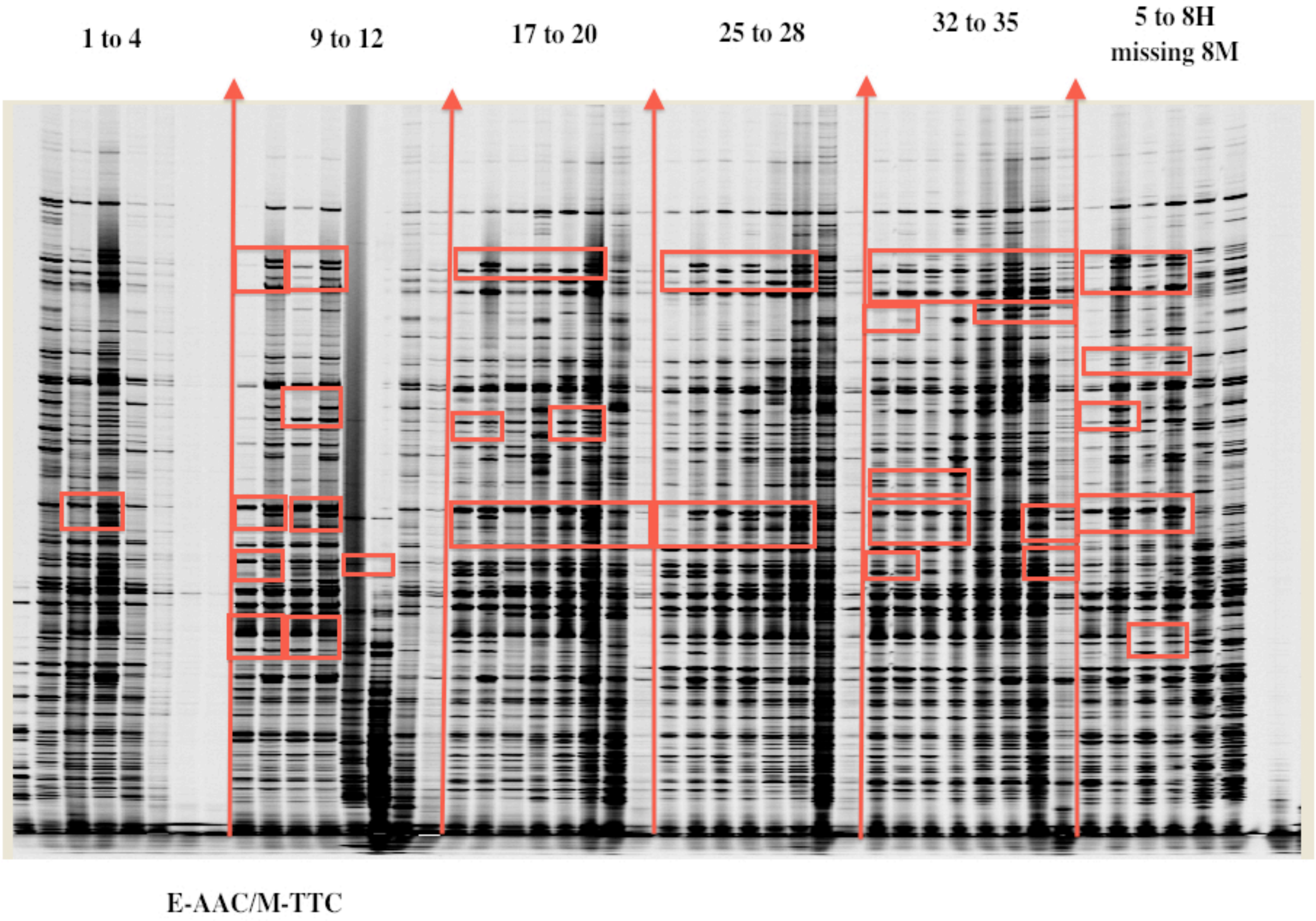
1 to 4
9 to 12
17 to 20
25 to 28
32 to 35
5 to 8H
missing 8M
E-AAC/M-TTC

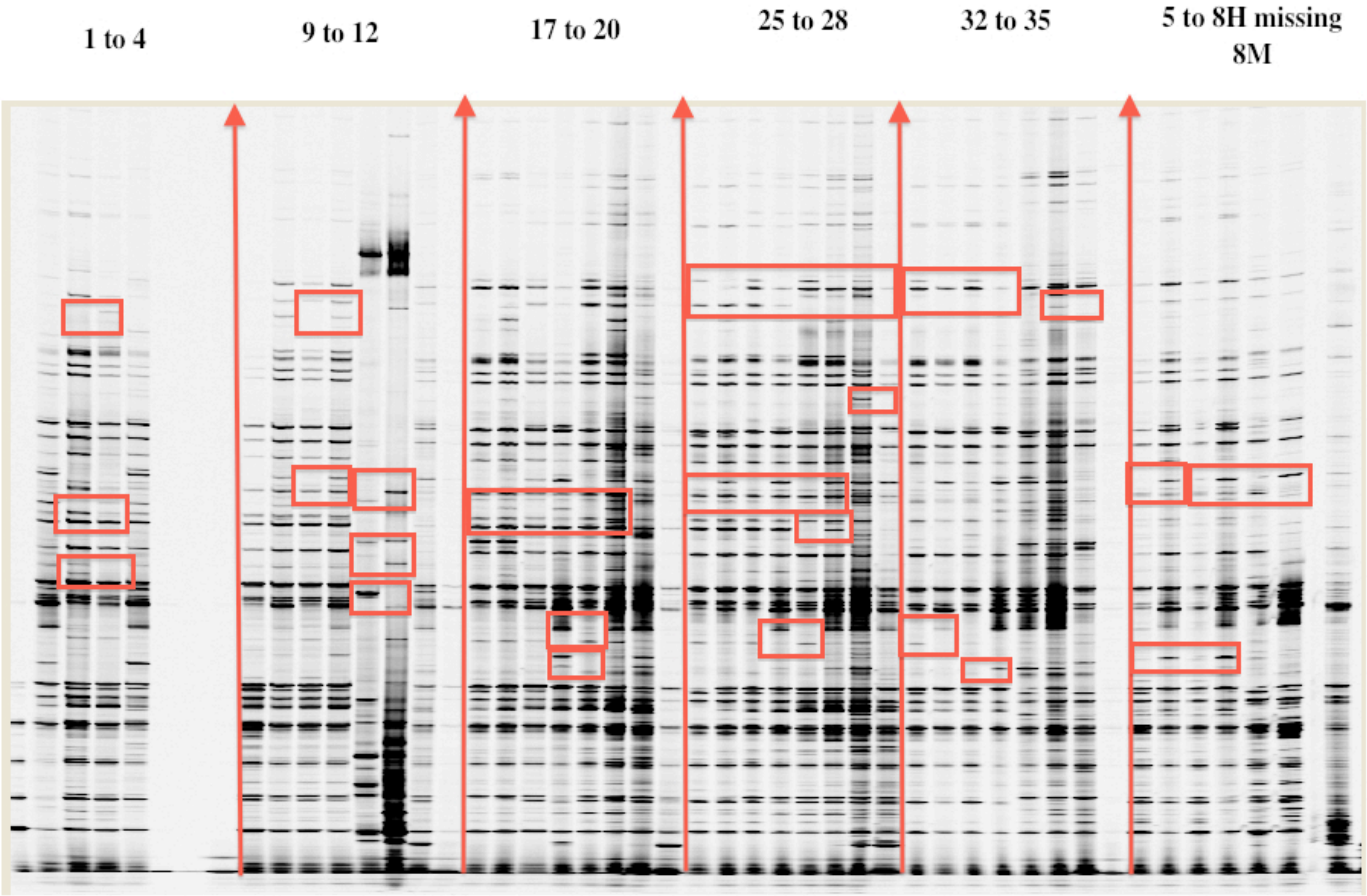

E-ACT/M-TCC

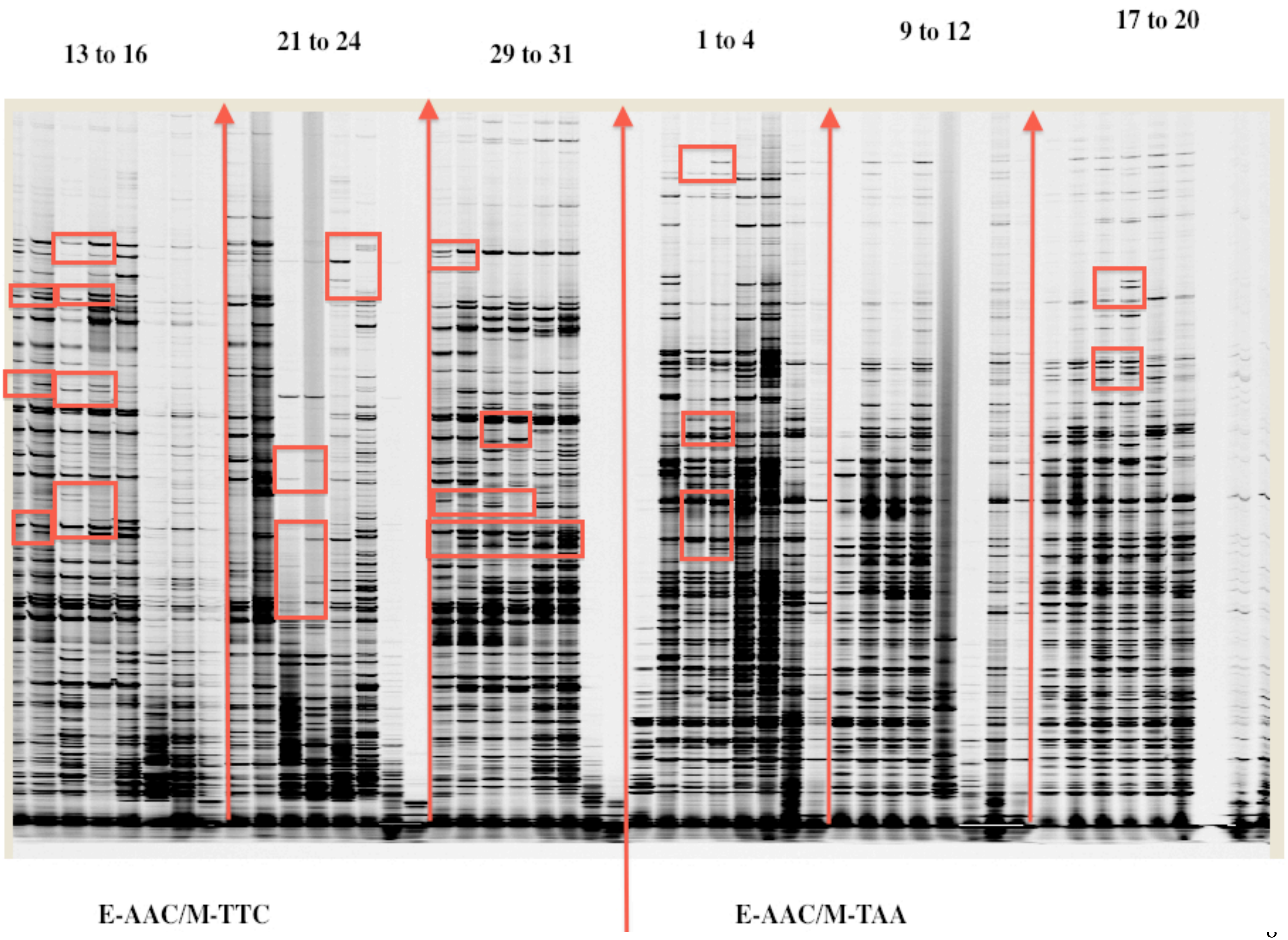
13 to 16
21 to 24
29 to 31
1 to 4
9 to 12
17 to 20
E-AAC/M-TTC
E-AAC/M-TAA

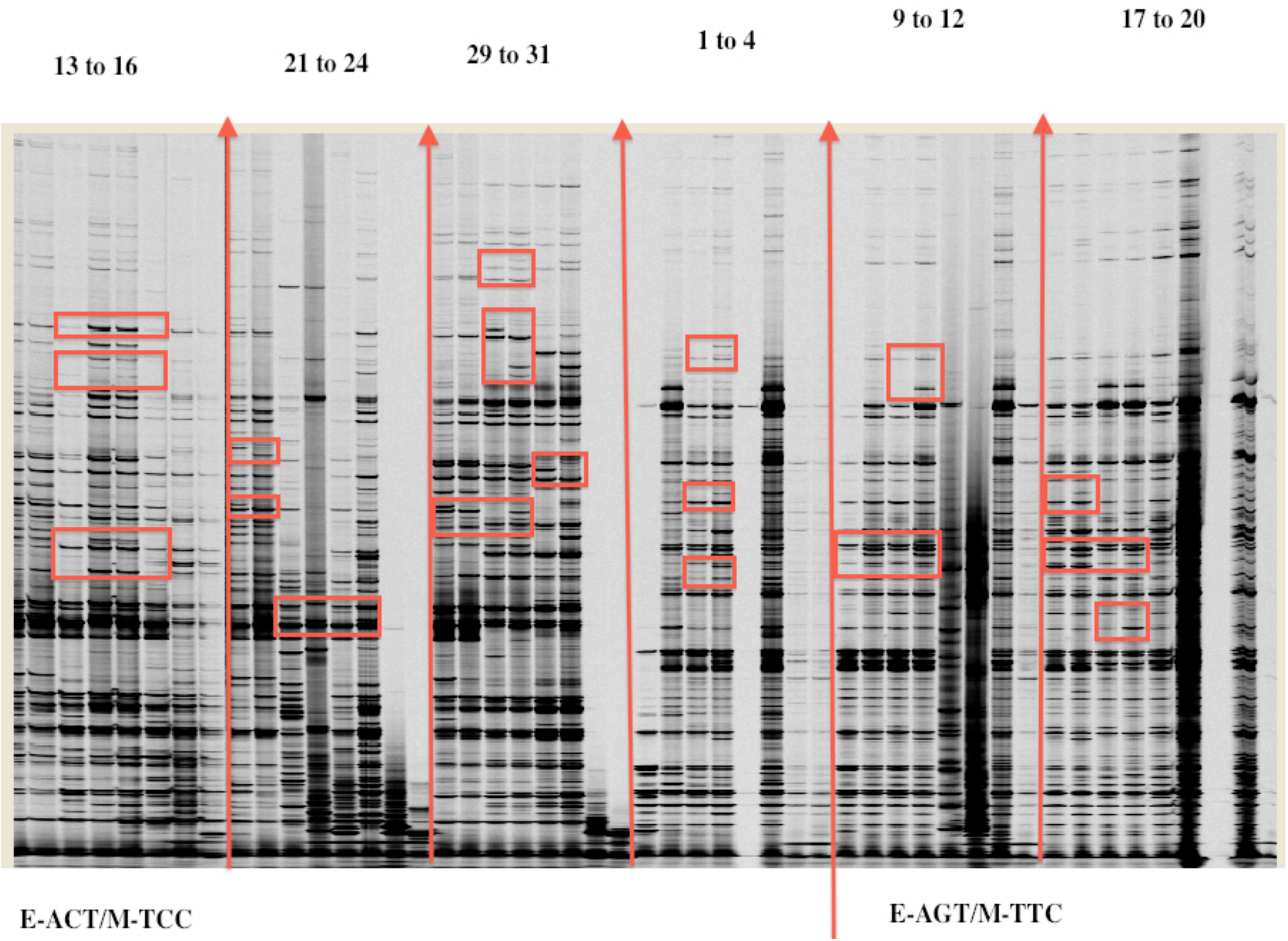
13 to 16
21 to 24
29 to 31
1 to 4
9 to 12
17 to 20
E-ACT/M-TCC
E-AGT/M-TTC

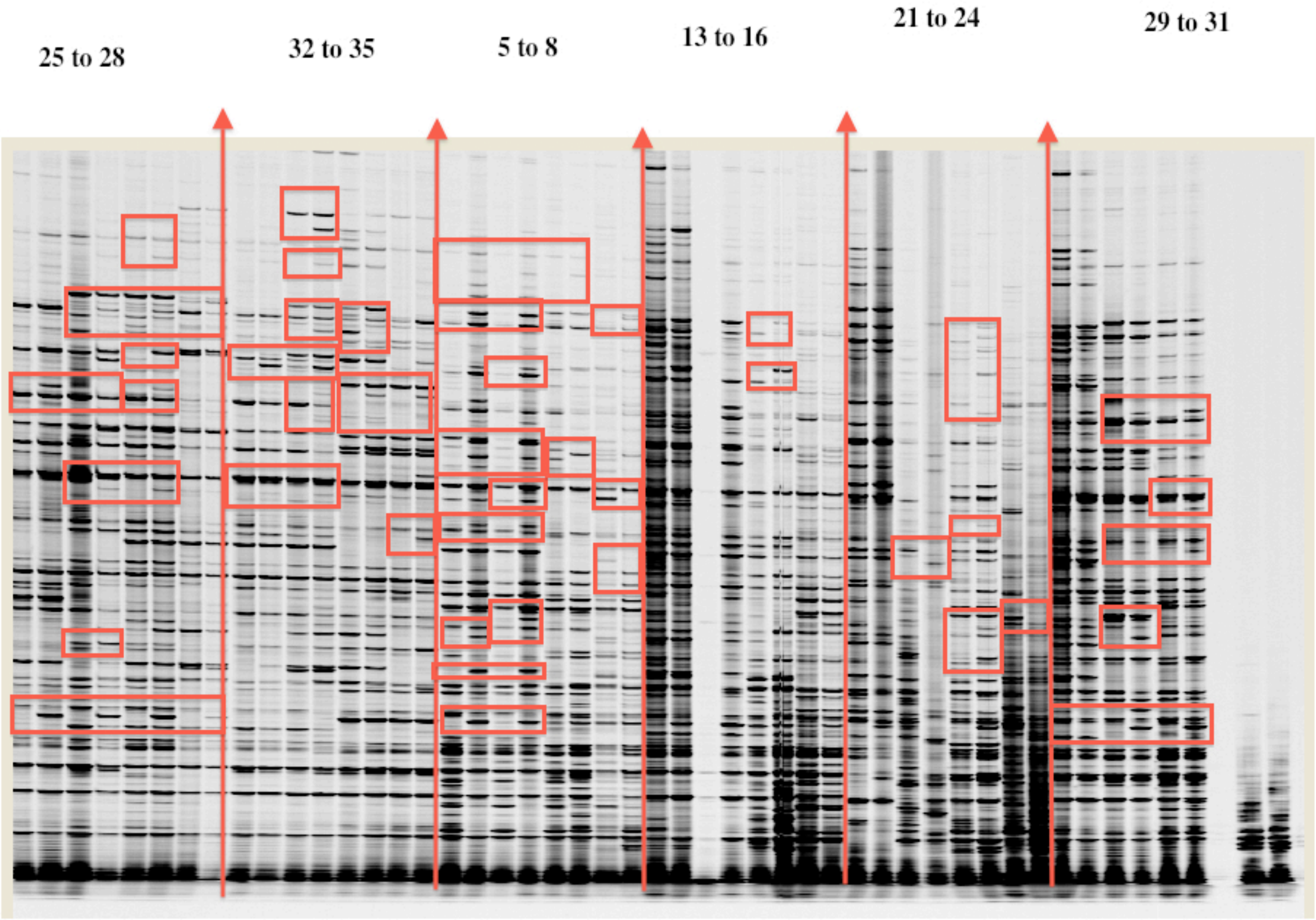

E-AAC/M-TAA

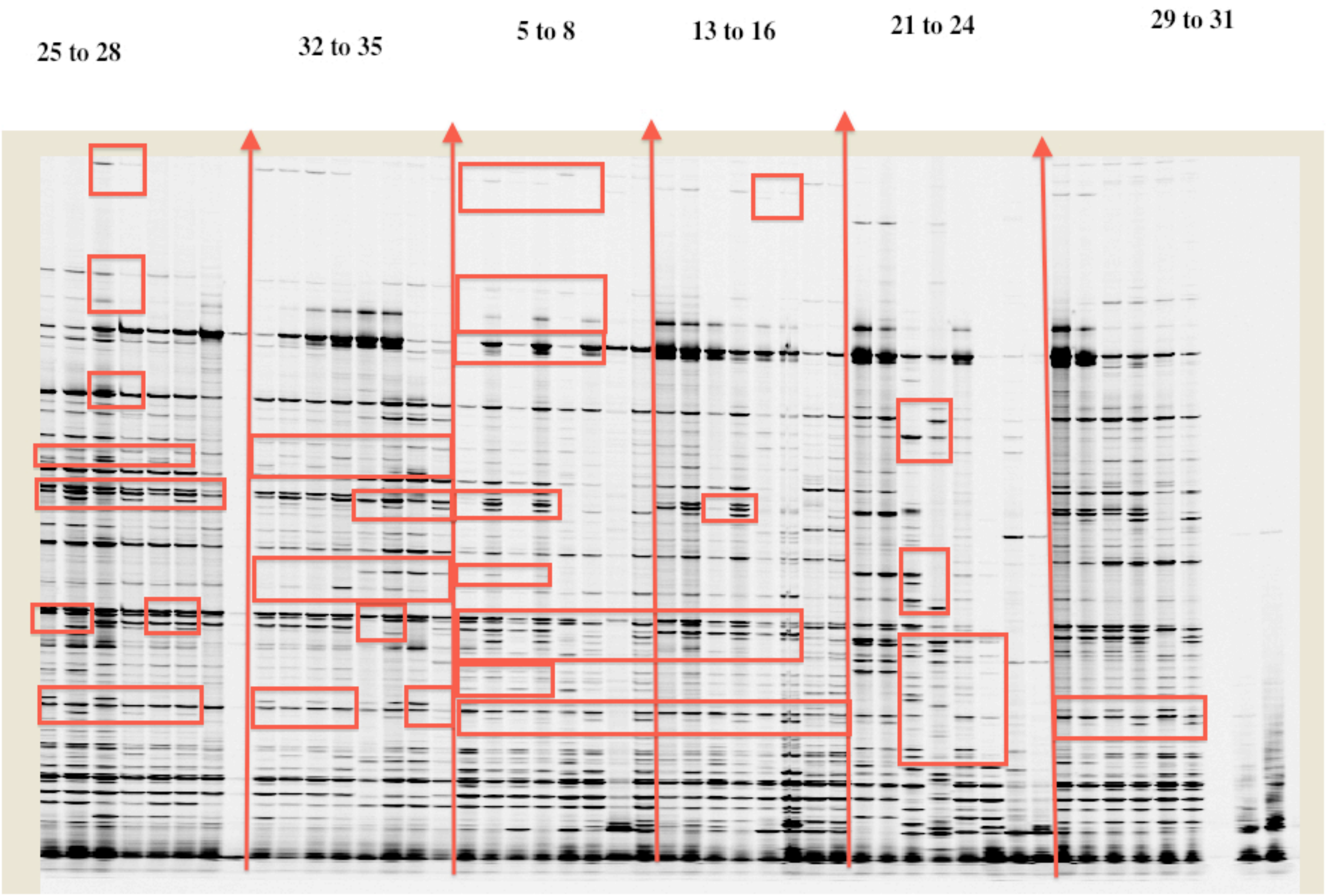

E-AGT/M-TTC

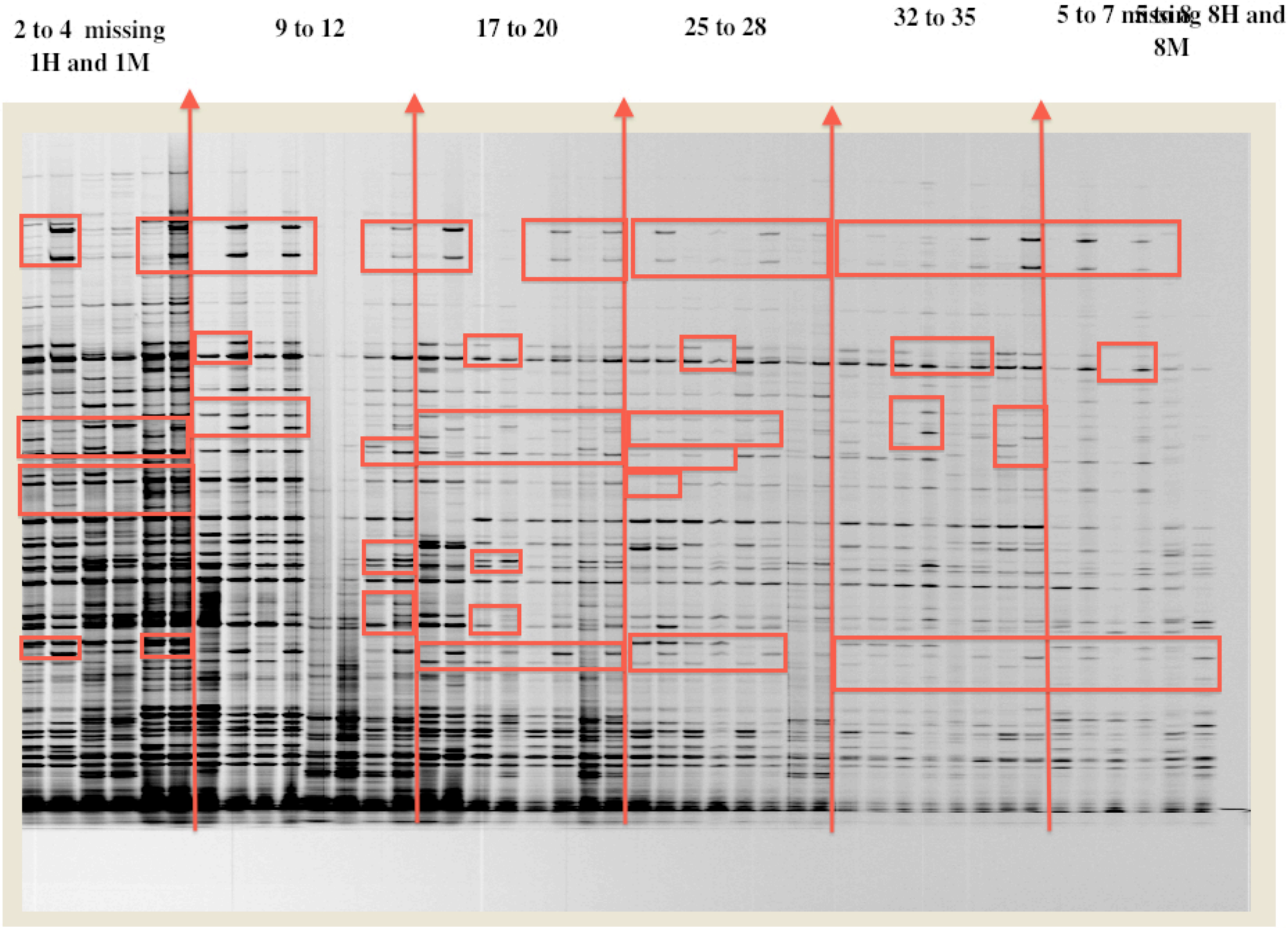
2 to 4 missing 1H and 1M
9 to 12
17 to 20
25 to 28
32 to 35
5 to 7 missing 8H and 8M
5 to 8

E-AAC/M-TCC

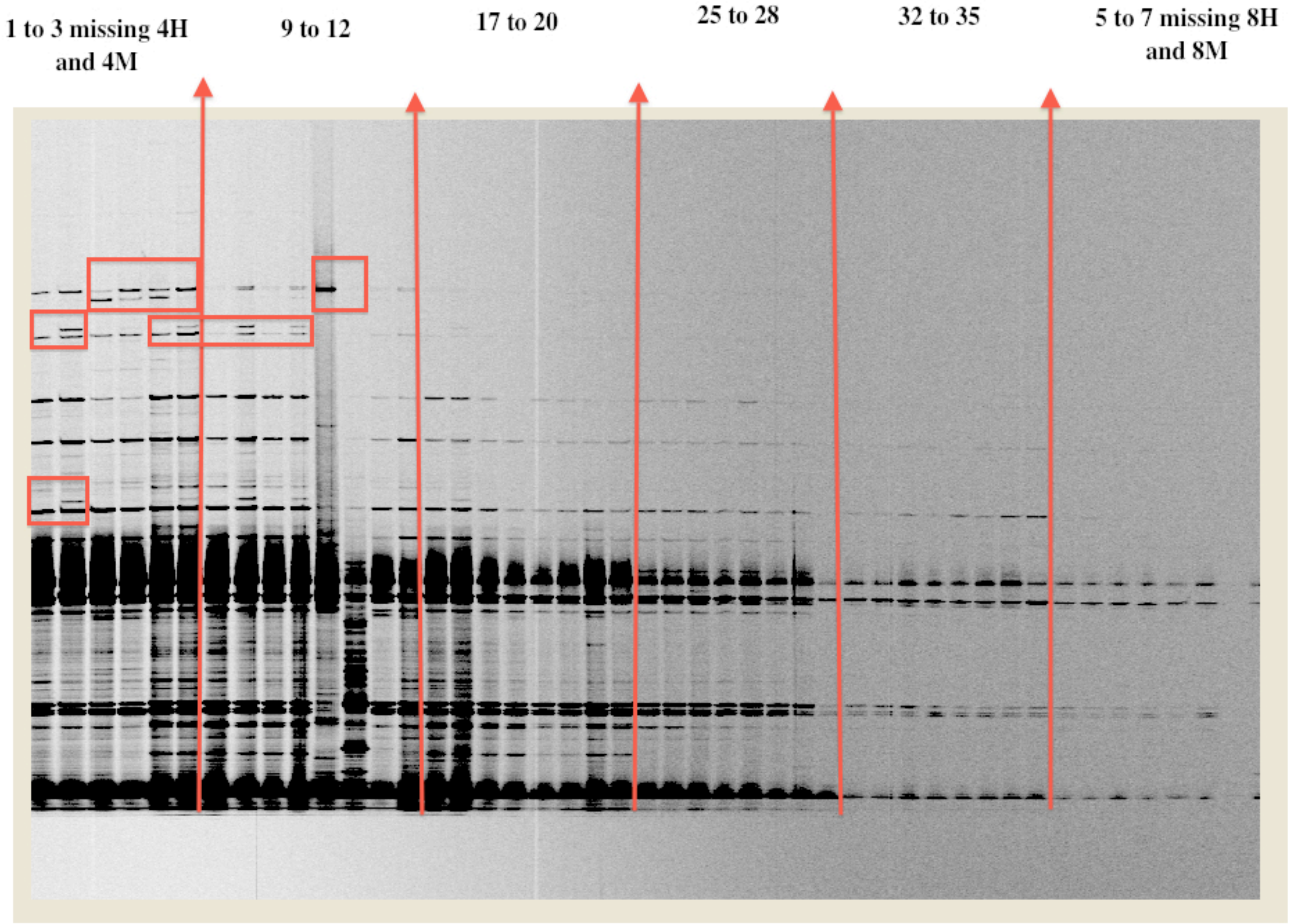
1 to 3 missing 4H and 4M
9 to 12
17 to 20
25 to 28
32 to 35
5 to 7 missing 8H and 8M

E-AGT/M-TAA

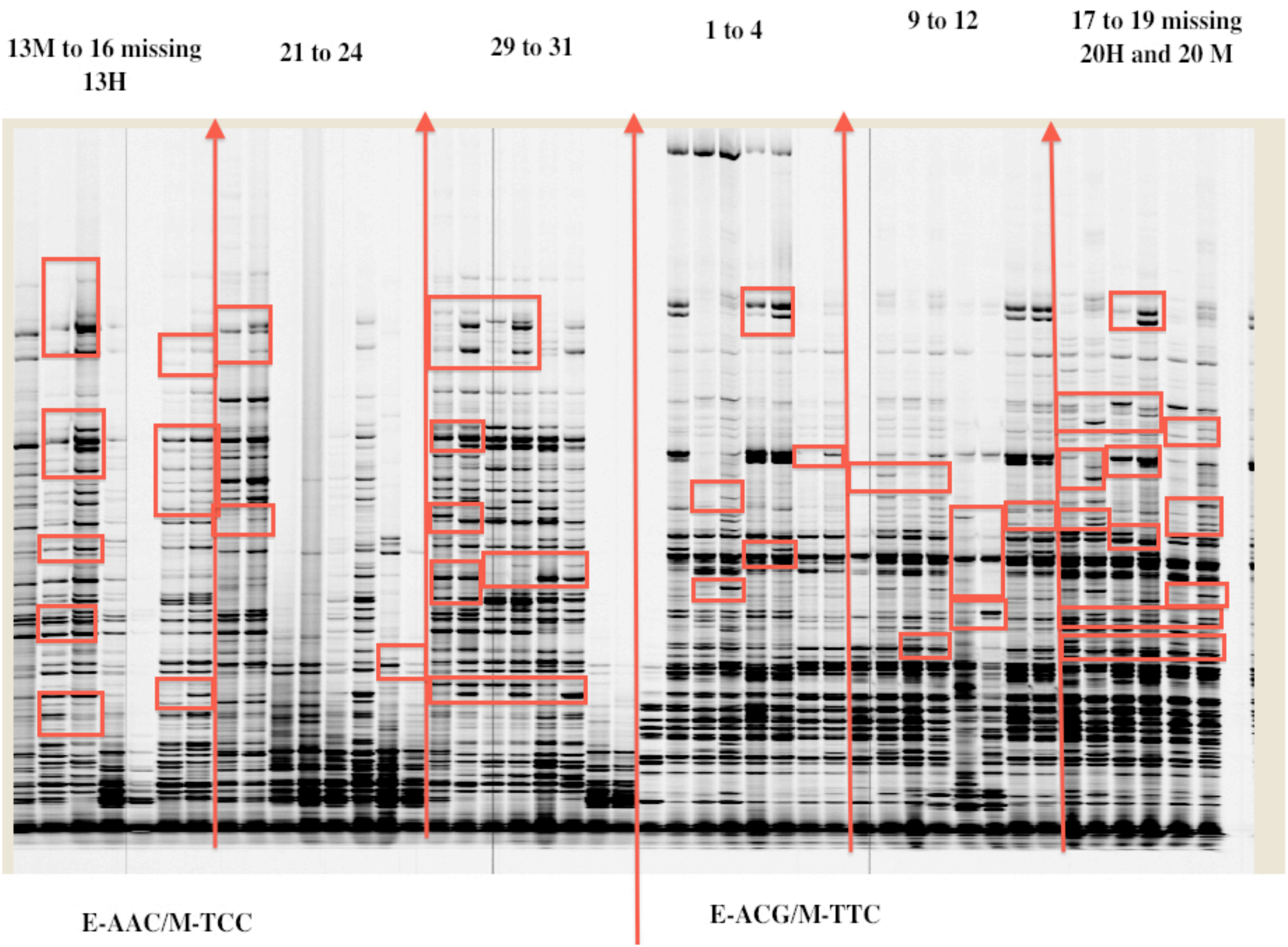
13M to 16 missing
13H
21 to 24
29 to 31
1 to 4
9 to 12
17 to 19 missing
20H and 20 M
E-AAC/M-TCC
E-ACG/M-TTC

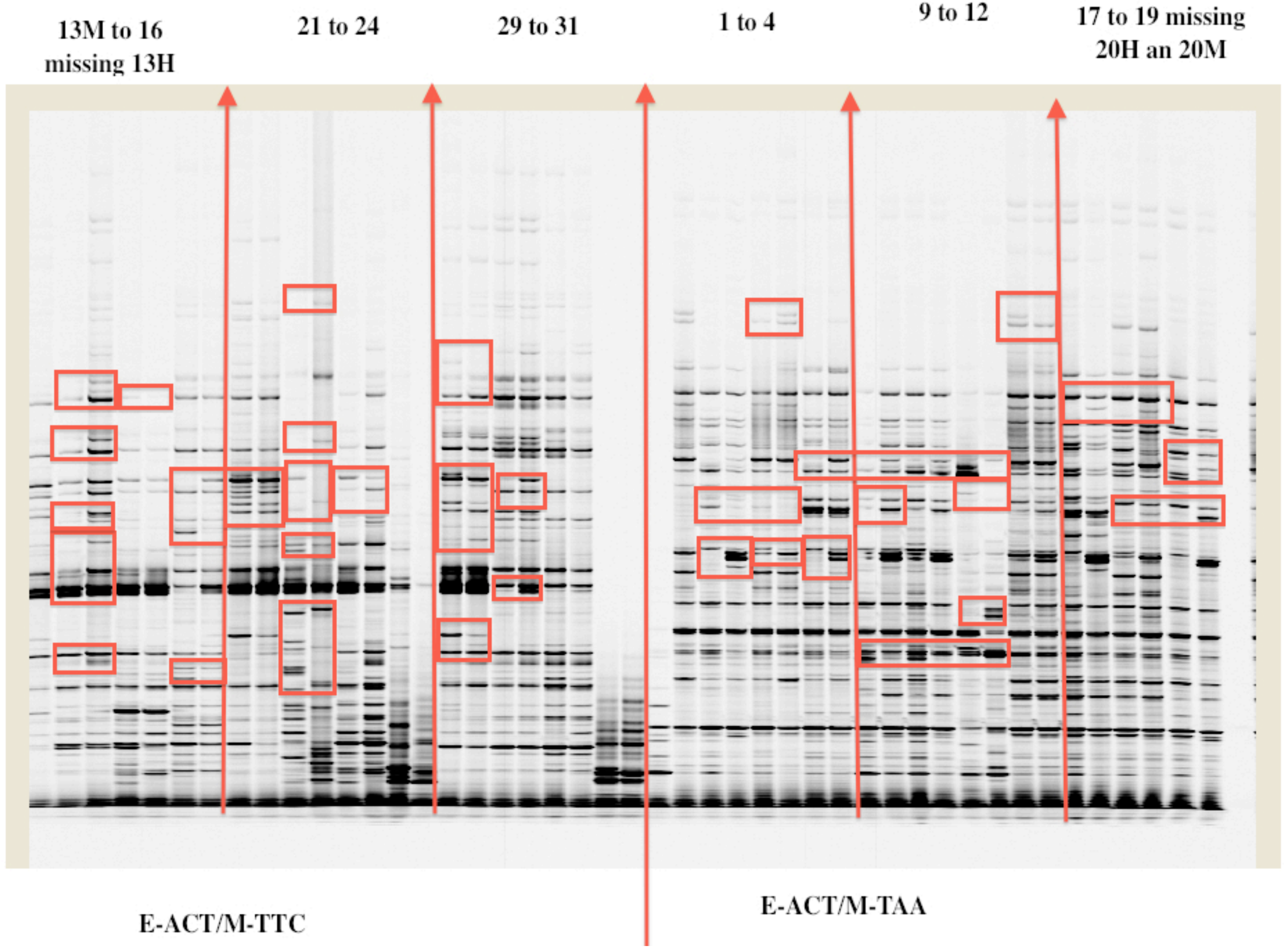
13M to 16
missing 13H
21 to 24
29 to 31
1 to 4
9 to 12
17 to 19 missing
20H an 20M
E-ACT/M-TTC
E-ACT/M-TAA

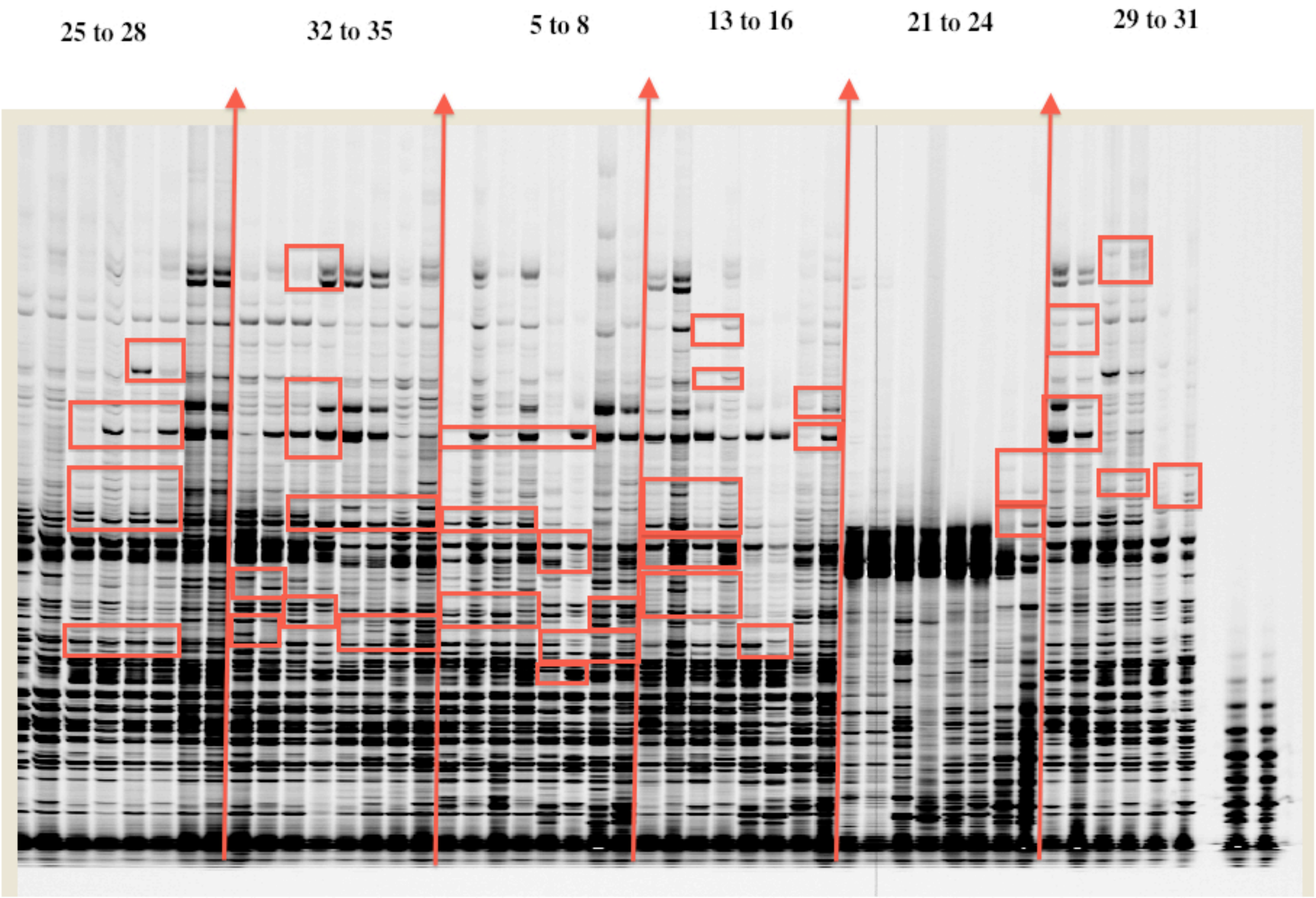

E-ACG/M-TTC

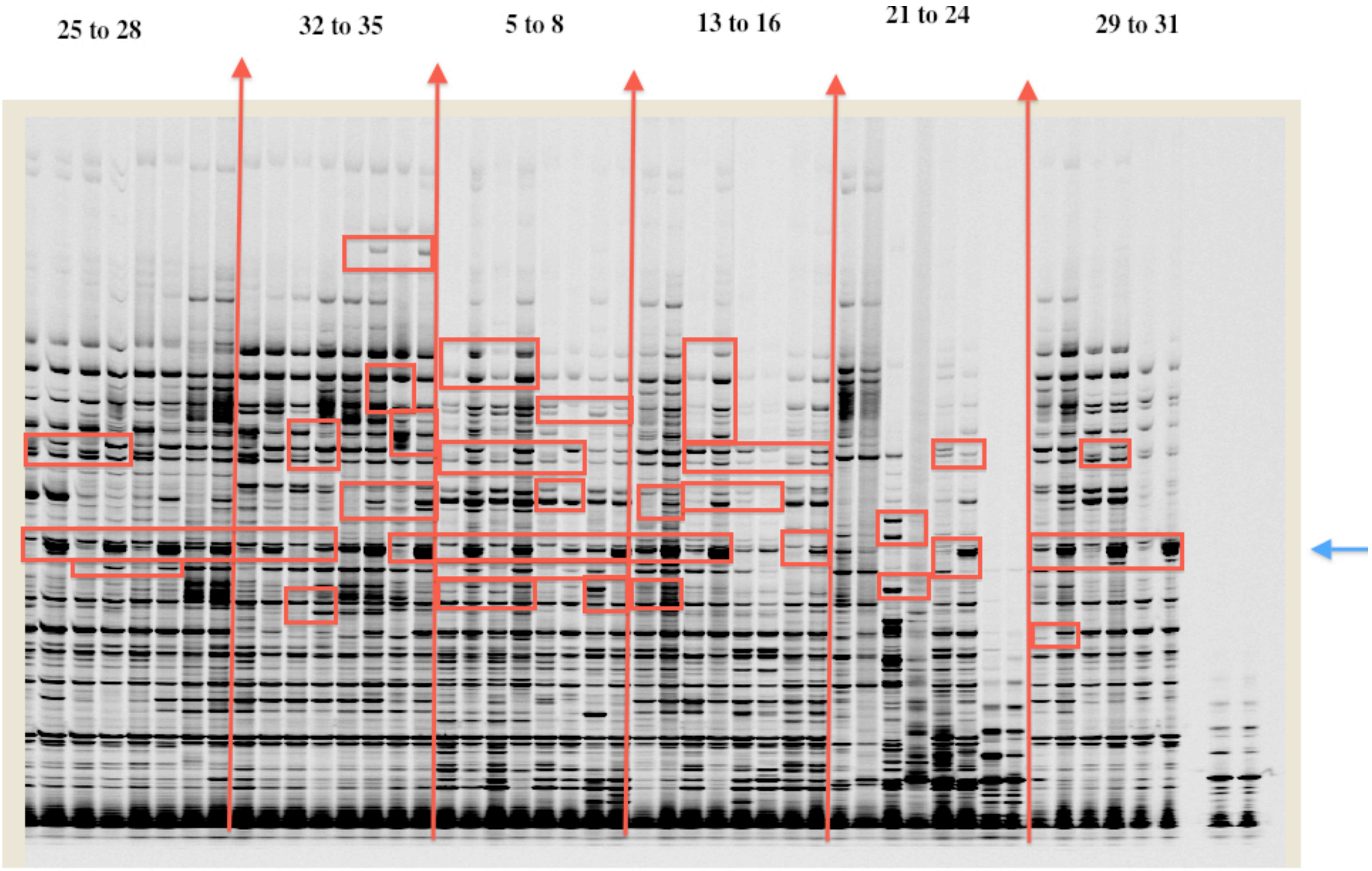

E-ACT/M-TAA

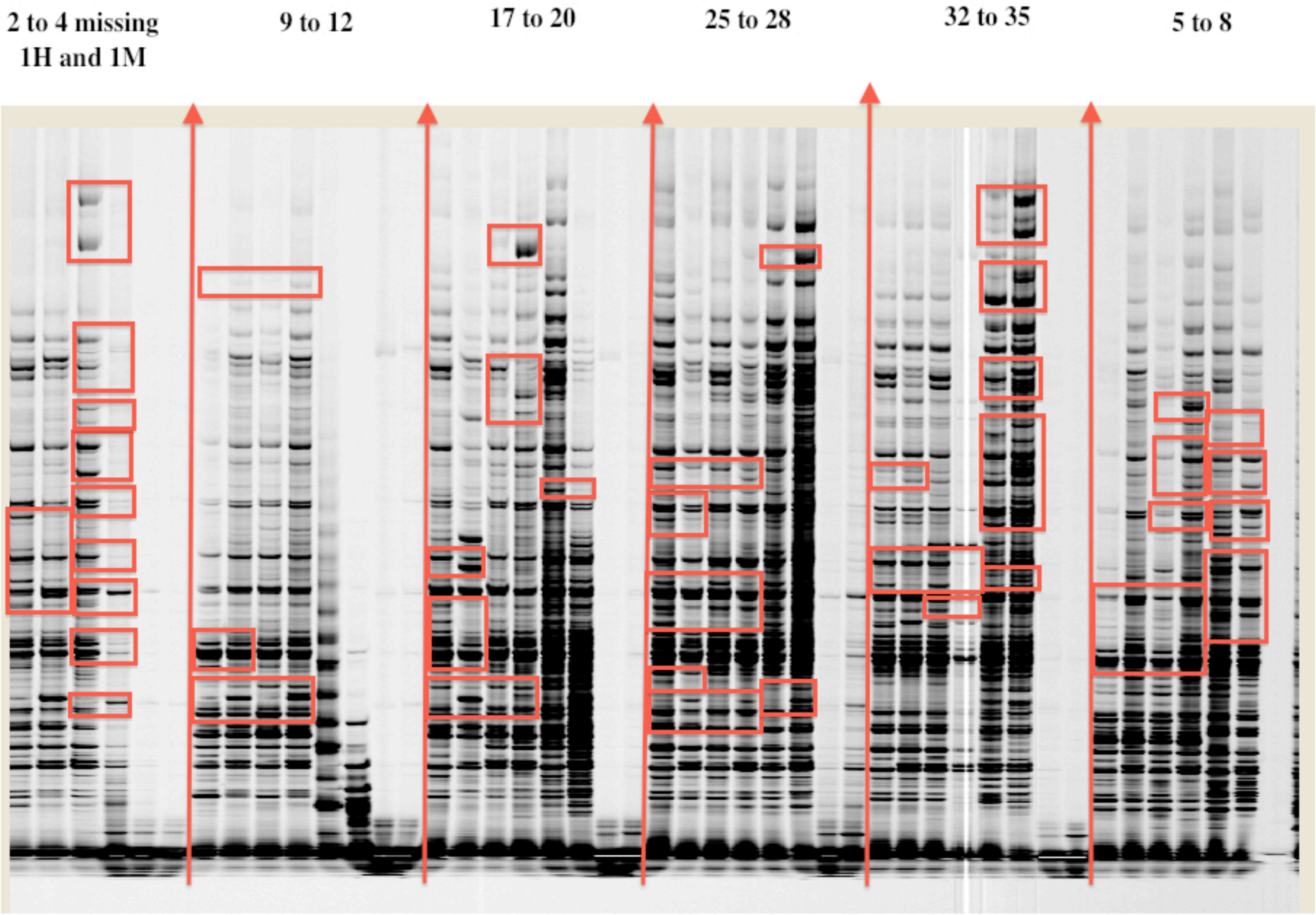

E-ACG/M-TAA

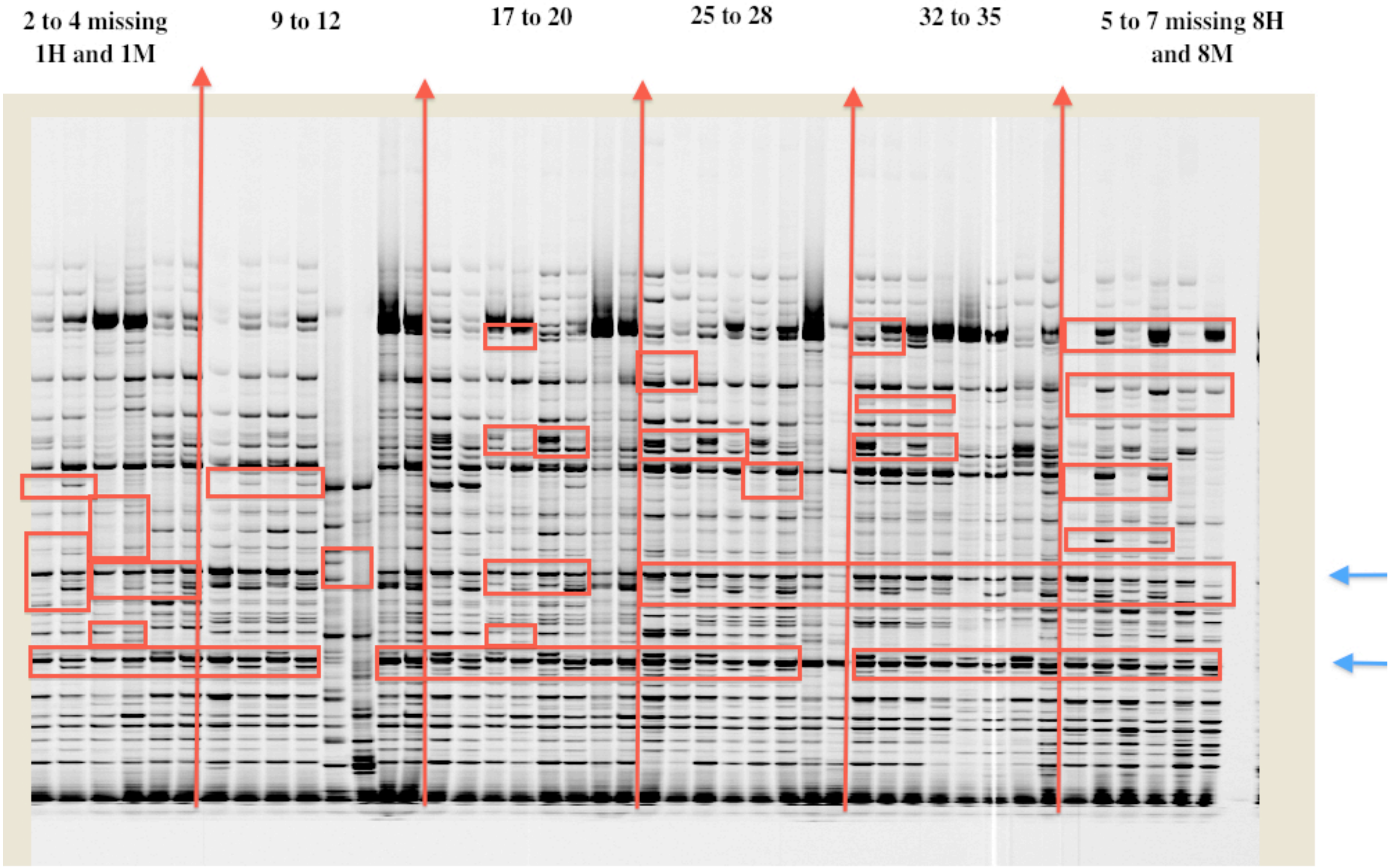
2 to 4 missing 1H and 1M
9 to 12
17 to 20
25 to 28
32 to 35
5 to 7 missing 8H and 8M

E-AGT/M-TCC

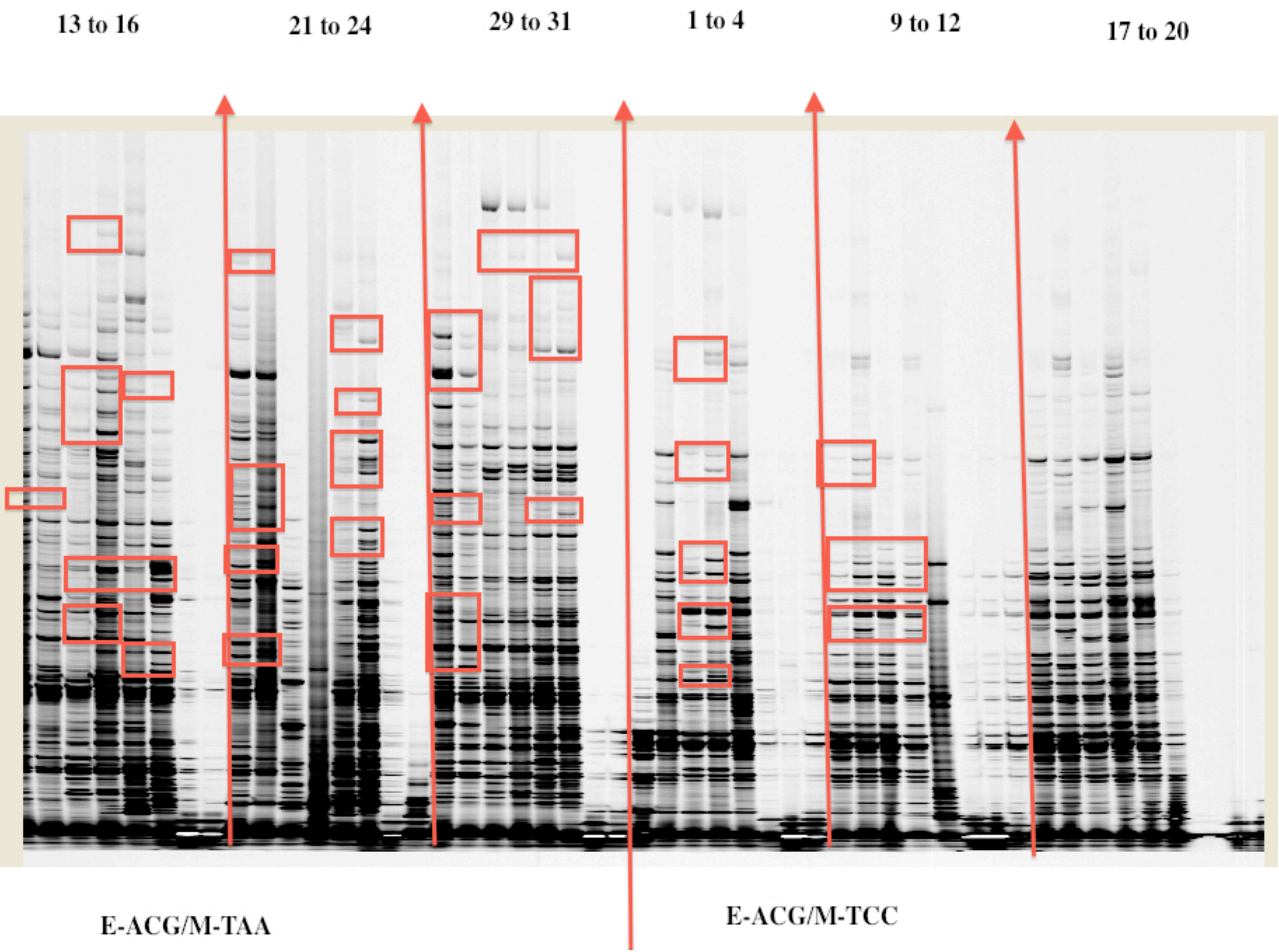

13 to 16
21 to 24
29 to 31
1 to 4
9 to 12
17 to 20
E-ACG/M-TAA
E-ACG/M-TCC

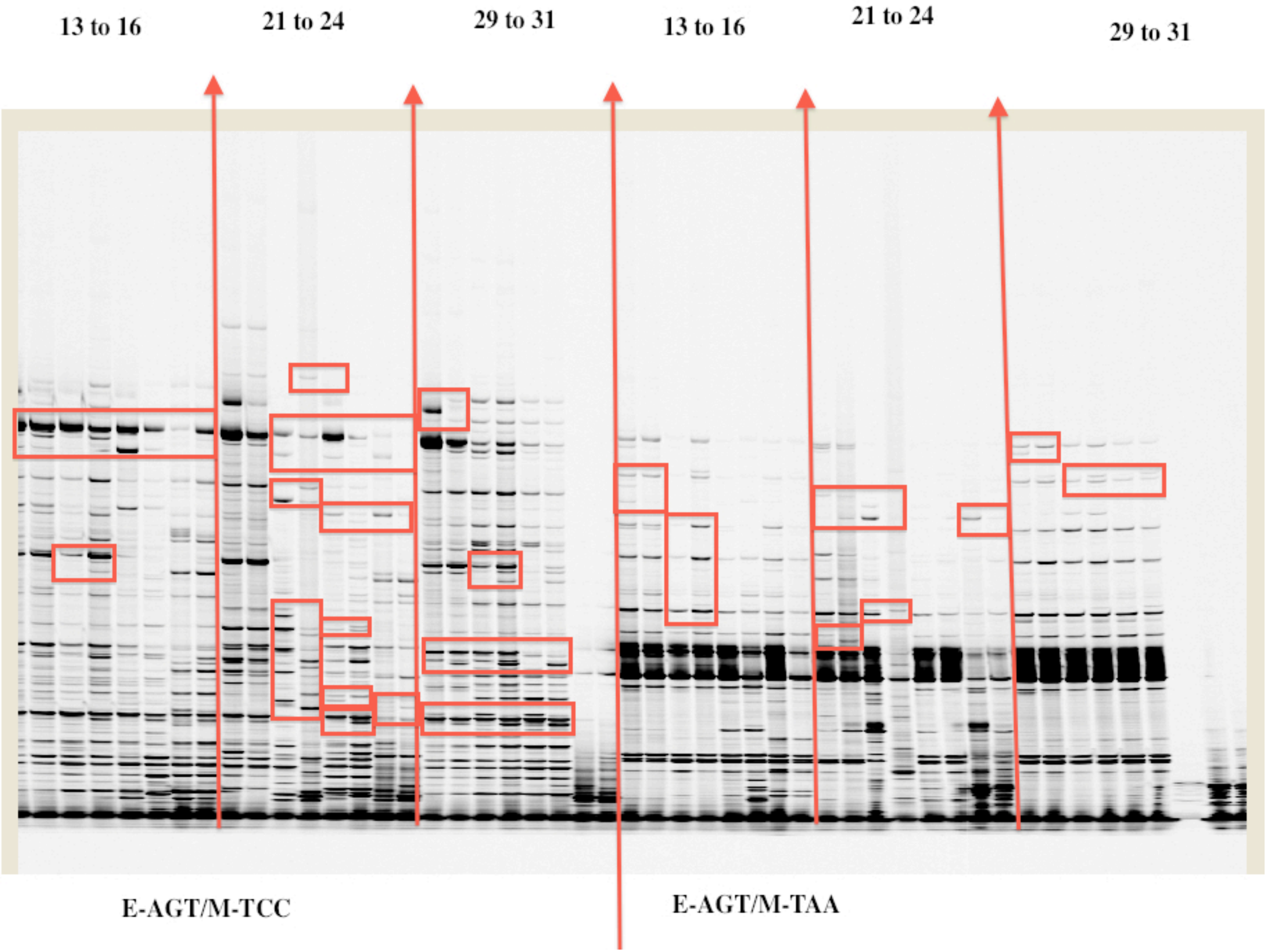
13 to 16
21 to 24
29 to 31
13 to 16
21 to 24
29 to 31
E-AGT/M-TCC
E-AGT/M-TAA

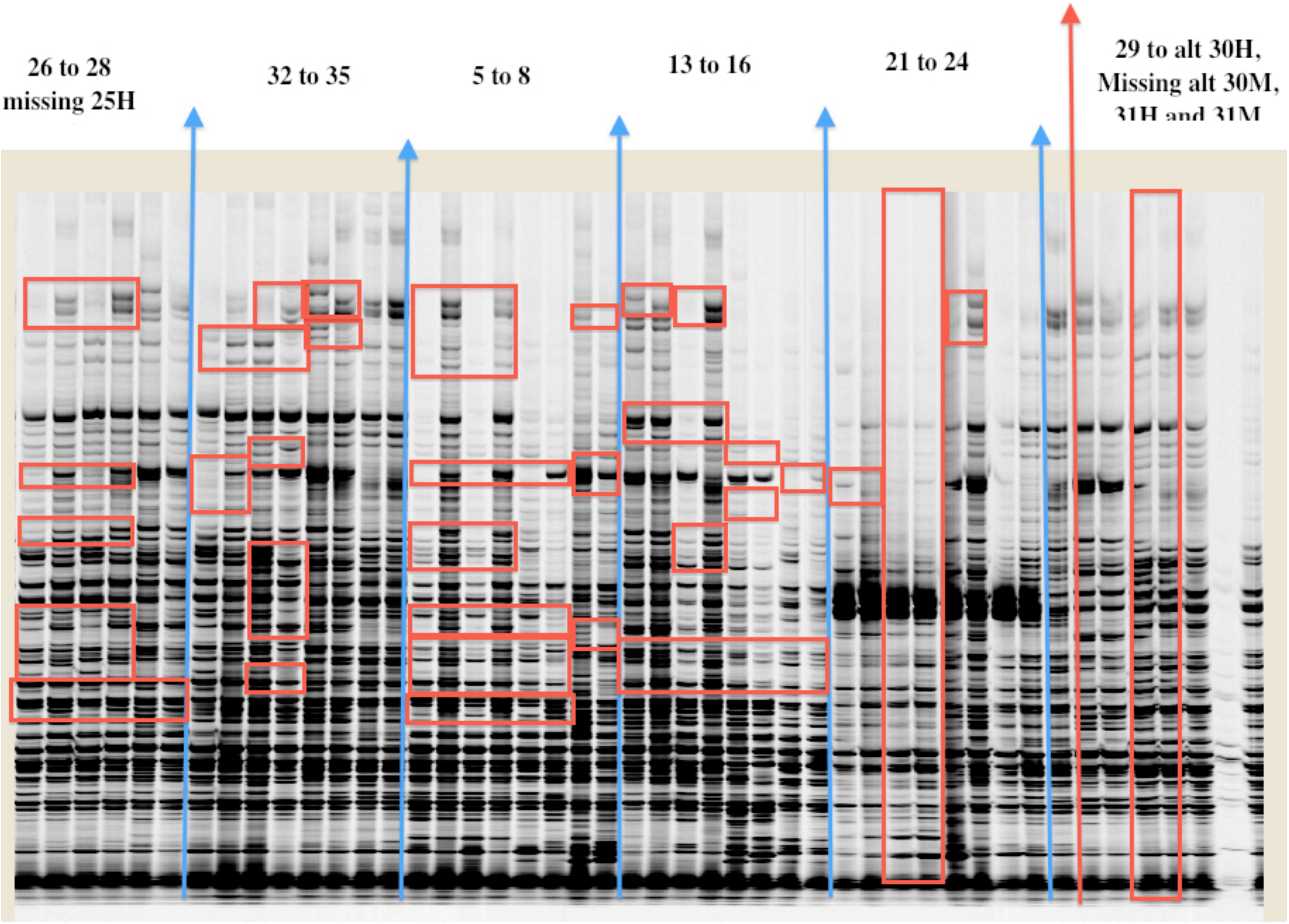
26 to 28
missing 25H
32 to 35
5 to 8
13 to 16
21 to 24
29 to alt 30H,
Missing alt 30M,

E-ACG/M-TCC

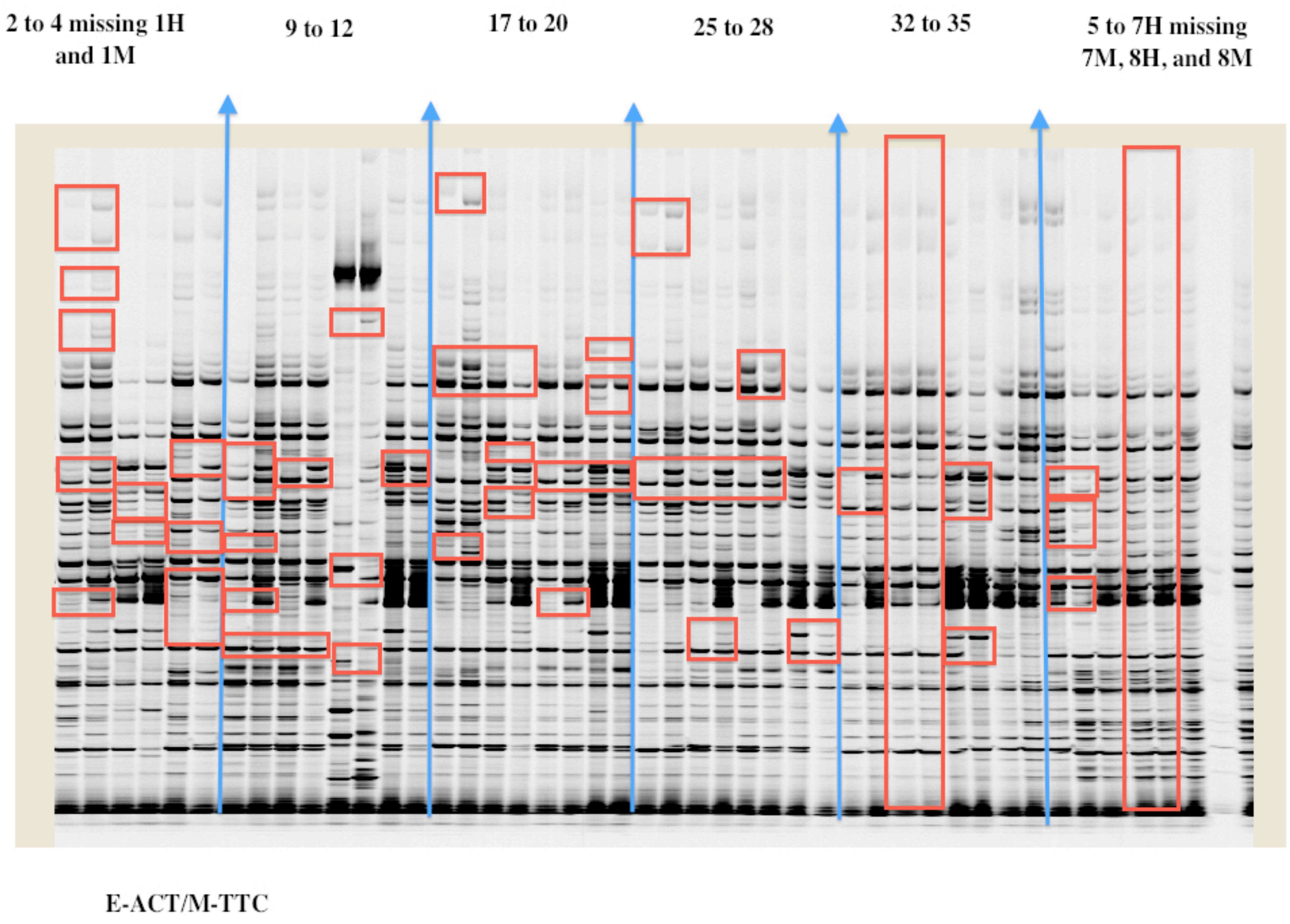
2 to 4 missing 1H and 1M
9 to 12
17 to 20
25 to 28
32 to 35
5 to 7H missing 7M, 8H, and 8M
E-ACT/M-TTC

Figure S1. Mosaic plot showing a departure from independence between BF-exhibition and DNA-(de)methylation, the shading in each of the four cells indicates that each cell exceeds the critical value of α=0.05 for significance difference between Pearson residuals, which reveals the pattern of lack of fit for a model suggesting total independence between BF-exhibition and DNA-(de)methylation.

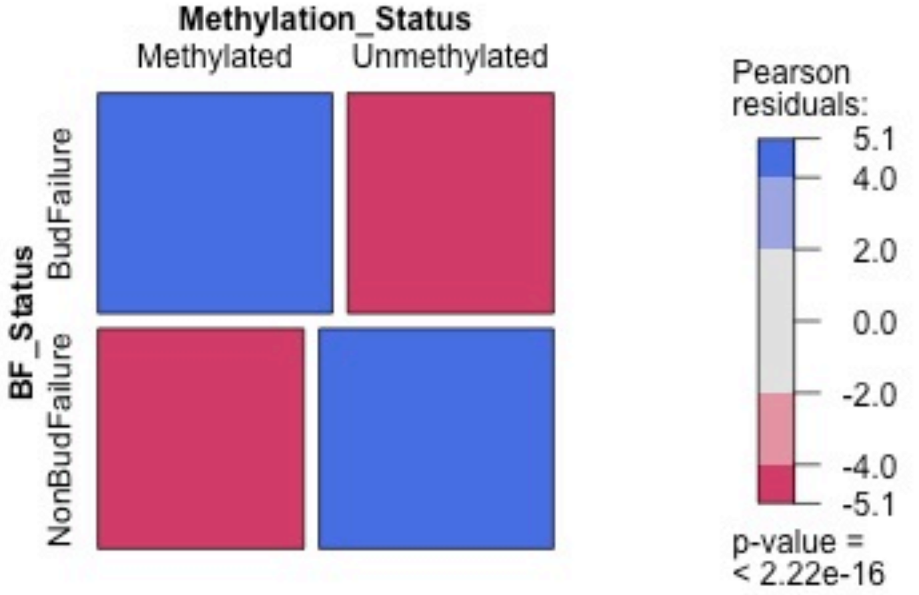

Supplementary Table S4: Kendall τ correlation coefficients to assess the concordance in patterns of DNA-(de)methylation

Genotype	by Genotype	Kendall τ	Prob>|τ|
Stukey6	Stukey5	0.5534	<.0001
Turkmen	Nonpareil	0.4334	<.0001
Winters	Stukey5	0.4175	<.0001
Winters	Stukey6	0.4048	<.0001
Nonpareil	Carmel	0.3799	<.0001
Winters	Mission	0.3381	<.0001
Winters	Nonpareil	0.3105	<.0001
Turkmen	Stukey5	0.3096	<.0001
Turkmen	Stukey6	0.306	<.0001
Stukey5	Nonpareil	0.2921	<.0001
Stukey6	Nonpareil	0.2765	<.0001
Turkmen	Carmel	0.2652	<.0001
Stukey5	Mission	0.2627	<.0001
Nonpareil	Mission	0.237	<.0001
Winters	Carmel	0.2266	<.0001
Stukey6	Mission	0.2244	<.0001
Stukey5	Carmel	0.2103	<.0001
Winters	Turkmen	0.1763	<.0001
Turkmen	Mission	0.1674	<.0001
Stukey6	Carmel	0.1666	<.0001
Mission	Carmel	0.1017	<.0001
**Average**		**0.2885**	

Table S5: Kendall τ correlation coefficients for the pairwise concordance in patterns of DNA-(de)methylation among the clonal sources considered in the present study. They are ordered in descending order with respect to the size of the Kendall τ.

Genotype	by Genotype	Kendall τ	Prob>|τ|
Winters_Browne	Winters_R11_1	0.6138	<.0001
Nonpareil_Arboretum	Nonpareil_Esparto1	0.5988	<.0001
Stukey_6_1	Stukey_5_2	0.5914	<.0001
Stukey_5_2	Stukey_5_1	0.5771	<.0001
Stukey_6_1	Winters_R11_1	0.4901	<.0001
Stukey_6_1	Stukey_5_1	0.4878	<.0001
Stukey_5_2	Winters_R11_1	0.4719	<.0001
Turkmen_Repo1	Nonpareil_Arboretum	0.4481	<.0001
Stukey_5_1	Winters_R11_1	0.447	<.0001
Stukey_5_2	Winters_Browne	0.4327	<.0001
Stukey_5_1	Winters_Browne	0.4231	<.0001
Stukey_6_1	Winters_Browne	0.4128	<.0001
Turkmen_Repo1	Nonpareil_Esparto1	0.3986	<.0001
Nonpareil_Arboretum	Carmel_PFS	0.394	<.0001
Nonpareil_PFS3	Nonpareil_PFS2	0.3779	<.0001
Carmel_ArbMarine	Carmel_PFS	0.368	<.0001
Nonpareil_Esparto1	Carmel_PFS	0.3617	<.0001
Stukey_6_2	Winters_Browne	0.3548	<.0001
Nonpareil_ArbMarine	Carmel_ArbMarine	0.3538	<.0001
Turkmen_Repo1 2	Nonpareil_PFS3	0.3446	<.0001
Nonpareil_PFS3	Nonpareil_PFS1	0.3346	<.0001
Mission_Wolfskill	Winters_R11_1	0.3342	<.0001
Turkmen_Repo1	Carmel_PFS	0.3281	<.0001
Stukey_6_2	Stukey_5_2	0.324	<.0001
Nonpareil_Arboretum	Nonpareil_ArbMarine	0.3235	<.0001
Turkmen_Repo1	Stukey_5_1	0.3211	<.0001
Stukey_6_2	Stukey_5_1	0.3161	<.0001
Stukey_6_2	Winters_R11_1	0.3121	<.0001
Stukey_6_2	Nonpareil_ArbMarine	0.3114	<.0001
Nonpareil_Esparto1	Carmel_Wolfskill	0.3057	<.0001
Stukey_6_2	Stukey_6_1	0.3012	<.0001
Stukey_6_1	Mission_Wolfskill	0.3003	<.0001
Nonpareil_Arboretum	Carmel_ArbMarine	0.2982	<.0001
Turkmen_Repo1	Nonpareil_ArbMarine	0.2935	<.0001
Mission_Wolfskill	Winters_Browne	0.2924	<.0001
Stukey_5_2	Mission_Wolfskill	0.2915	<.0001
Nonpareil_ArbMarine	Nonpareil_Esparto1	0.2847	<.0001
Stukey_5_1	Mission_Wolfskill	0.2723	<.0001
Turkmen_Repo1 2	Stukey_6_2	0.2664	<.0001

Stukey_5_1	Nonpareil_Arboretum	0.2636	<.0001
Nonpareil_Esparto2	Nonpareil_Esparto1	0.2612	<.0001
Turkmen_Repo1 2	Nonpareil_PFS2	0.2609	<.0001
Nonpareil_Arboretum	Winters_Browne	0.2544	<.0001
Turkmen_Repo1	Stukey_5_2	0.2517	<.0001
Nonpareil_Esparto1	Carmel_ArbMarine	0.2516	<.0001
Stukey_5_2	Carmel_PFS	0.2507	<.0001
Nonpareil_ArbMarine	Winters_Browne	0.2482	<.0001
Turkmen_Repo1 2	Nonpareil_ArbMarine	0.2451	<.0001
Nonpareil_ArbMarine	Carmel_PFS	0.2441	<.0001
Stukey_5_1	Carmel_PFS	0.2432	<.0001
Nonpareil_PFS3	Mission_Wolfskill	0.2421	<.0001
Stukey_5_2	Nonpareil_PFS2	0.2406	<.0001
Nonpareil_PFS2	Nonpareil_PFS1	0.2404	<.0001
Nonpareil_Arboretum	Carmel_Wolfskill	0.2401	<.0001
Carmel_PFS	Carmel_Wolfskill	0.2351	<.0001
Nonpareil_PFS2	Nonpareil_Esparto1	0.235	<.0001
Stukey_6_1	Carmel_PFS	0.2343	<.0001
Nonpareil_Arboretum	Nonpareil_Esparto2	0.2335	<.0001
Stukey_6_1	Nonpareil_PFS1	0.2317	<.0001
Carmel_PFS	Winters_Browne	0.2314	<.0001
Stukey_5_2	Nonpareil_PFS3	0.231	<.0001
Nonpareil_Arboretum	Nonpareil_PFS3	0.2253	<.0001
Stukey_5_2	Nonpareil_Arboretum	0.2253	<.0001
Nonpareil_Esparto2	Winters_R11_2	0.2251	<.0001
Nonpareil_Arboretum	Winters_R11_1	0.2247	<.0001
Turkmen_Repo1	Stukey_6_1	0.2247	<.0001
Mission_PFS	Mission_Wolfskill	0.2242	<.0001
Nonpareil_Esparto2	Carmel_Wolfskill	0.2238	<.0001
Nonpareil_PFS1	Mission_PFS	0.223	<.0001
Nonpareil_ArbMarine	Nonpareil_Esparto2	0.2223	<.0001
Stukey_5_1	Nonpareil_Esparto1	0.222	<.0001
Nonpareil_PFS3	Mission_PFS	0.2186	<.0001
Nonpareil_Arboretum	Nonpareil_PFS2	0.2184	<.0001
Stukey_5_2	Nonpareil_PFS1	0.2176	<.0001
Stukey_5_1	Nonpareil_PFS3	0.2153	<.0001
Nonpareil_PFS1	Winters_R11_1	0.2136	<.0001
Nonpareil_PFS3	Nonpareil_Esparto1	0.2135	<.0001
Turkmen_Repo1	Winters_R11_1	0.2116	<.0001
Turkmen_Repo1 2	Nonpareil_Esparto1	0.2108	<.0001
Stukey_6_1	Nonpareil_Arboretum	0.21	<.0001
Stukey_5_1	Nonpareil_PFS1	0.209	<.0001
Mission_Wolfskill	Carmel_PFS	0.2049	<.0001

Nonpareil_PFS1	Mission_Wolfskill	0.2018	<.0001
Carmel_PFS	Winters_R11_1	0.2016	<.0001
Turkmen_Repo1	Nonpareil_Esparto2	0.2004	<.0001
Turkmen_Repo1	Winters_Browne	0.1979	<.0001
Turkmen_Repo1	Carmel_ArbMarine	0.1974	<.0001
Turkmen_Repo1 2	Nonpareil_Arboretum	0.196	<.0001
Nonpareil_Esparto1	Winters_Browne	0.1953	<.0001
Carmel_Wolfskill	Winters_R11_2	0.1949	<.0001
Nonpareil_PFS1	Winters_Browne	0.1943	<.0001
Turkmen_Repo1	Carmel_Wolfskill	0.1926	<.0001
Turkmen_Repo1 2	Winters_Browne	0.1926	<.0001
Nonpareil_PFS3	Winters_PFS	0.1911	<.0001
Turkmen_Repo1	Nonpareil_PFS2	0.1902	<.0001
Carmel_ArbMarine	Carmel_Wolfskill	0.1897	<.0001
Turkmen_Repo1 2	Stukey_5_1	0.1897	<.0001
Nonpareil_PFS2	Carmel_Wolfskill	0.1896	<.0001
Nonpareil_PFS2	Nonpareil_Esparto2	0.1847	<.0001
Nonpareil_ArbMarine	Winters_R11_1	0.1832	<.0001
Nonpareil_Esparto1	Winters_R11_2	0.1811	<.0001
Nonpareil_Esparto1	Mission_Wolfskill	0.1799	<.0001
Stukey_6_1	Nonpareil_PFS3	0.1794	<.0001
Stukey_5_1	Nonpareil_ArbMarine	0.1793	<.0001
Turkmen_Repo1 2	Winters_R11_1	0.1782	<.0001
Nonpareil_PFS2	Winters_PFS	0.1774	<.0001
Turkmen_Repo1 2	Mission_Wolfskill	0.1764	<.0001
Mission_PFS	Winters_R11_1	0.1761	<.0001
Nonpareil_PFS3	Winters_R11_1	0.176	<.0001
Stukey_5_2	Mission_PFS	0.1755	<.0001
Carmel_ArbMarine	Winters_Browne	0.1749	<.0001
Stukey_6_2	Nonpareil_Esparto1	0.1744	<.0001
Nonpareil_Esparto1	Winters_R11_1	0.1737	<.0001
Nonpareil_Arboretum	Mission_Wolfskill	0.1732	<.0001
Nonpareil_PFS2	Carmel_PFS	0.1723	<.0001
Stukey_6_1	Nonpareil_PFS2	0.1722	<.0001
Turkmen_Repo1 2	Nonpareil_Esparto2	0.1715	<.0001
Stukey_5_1	Mission_PFS	0.1708	<.0001
Turkmen_Repo1	Mission_Wolfskill	0.1707	<.0001
Nonpareil_Arboretum	Winters_PFS	0.1677	<.0001
Nonpareil_PFS3	Winters_R11_2	0.1674	<.0001
Turkmen_Repo1	Stukey_6_2	0.1672	<.0001
Stukey_6_2	Nonpareil_Arboretum	0.1671	<.0001
Stukey_6_1	Nonpareil_Esparto1	0.1656	<.0001
Turkmen_Repo1	Nonpareil_PFS3	0.1636	<.0001

Nonpareil_PFS2	Mission_Wolfskill	0.1631	<.0001
Stukey_6_2	Carmel_ArbMarine	0.1602	<.0001
Stukey_6_2	Mission_Wolfskill	0.1602	<.0001
Turkmen_Repo1 2	Nonpareil_PFS1	0.1582	<.0001
Stukey_5_2	Nonpareil_Esparto1	0.1566	<.0001
Nonpareil_PFS3	Carmel_PFS	0.1561	<.0001
Carmel_ArbMarine	Winters_R11_1	0.1559	<.0001
Nonpareil_PFS3	Winters_Browne	0.1558	<.0001
Turkmen_Repo1 2	Turkmen_Repo1	0.1551	<.0001
Turkmen_Repo1 2	Winters_PFS	0.1546	<.0001
Turkmen_Repo1 2	Carmel_ArbMarine	0.1546	<.0001
Nonpareil_PFS1	Winters_PFS	0.1532	<.0001
Stukey_6_2	Nonpareil_PFS2	0.1526	<.0001
Turkmen_Repo1 2	Stukey_5_2	0.1518	<.0001
Nonpareil_PFS2	Winters_Browne	0.1493	<.0001
Stukey_5_1	Carmel_ArbMarine	0.146	<.0001
Stukey_6_2	Mission_PFS	0.143	<.0001
Winters_Browne	Winters_PFS	0.1422	<.0001
Stukey_5_2	Nonpareil_ArbMarine	0.1417	<.0001
Nonpareil_PFS3	Carmel_Wolfskill	0.1414	<.0001
Mission_PFS	Winters_Browne	0.1408	<.0001
Stukey_6_2	Nonpareil_Esparto2	0.1402	<.0001
Stukey_6_1	Nonpareil_ArbMarine	0.1388	<.0001
Turkmen_Repo1 2	Carmel_PFS	0.1388	<.0001
Turkmen_Repo1 2	Winters_R11_2	0.1361	<.0001
Stukey_5_1	Nonpareil_PFS2	0.1358	<.0001
Stukey_5_2	Carmel_ArbMarine	0.134	<.0001
Nonpareil_Esparto1	Winters_PFS	0.1278	<.0001
Stukey_6_2	Winters_PFS	0.1267	<.0001
Turkmen_Repo1	Nonpareil_PFS1	0.1241	<.0001
Mission_Wolfskill	Winters_PFS	0.1233	<.0001
Turkmen_Repo1 2	Mission_PFS	0.1223	<.0001
Nonpareil_PFS2	Winters_R11_1	0.1216	<.0001
Stukey_6_1	Mission_PFS	0.1207	<.0001
Nonpareil_Arboretum	Nonpareil_PFS1	0.1192	<.0001
Nonpareil_ArbMarine	Carmel_Wolfskill	0.1166	<.0001
Stukey_6_2	Carmel_PFS	0.1158	0.0001
Carmel_Wolfskill	Winters_PFS	0.1155	0.0001
Turkmen_Repo1	Winters_PFS	0.1132	0.0001
Turkmen_Repo1 2	Carmel_Wolfskill	0.1115	0.0002
Nonpareil_Arboretum	Mission_PFS	0.1112	0.0002
Carmel_PFS	Winters_PFS	0.111	0.0002
Mission_PFS	Winters_PFS	0.1099	0.0002

Turkmen_Repo1 2	Stukey_6_1	0.1088	0.0003
Nonpareil_Arboretum	Winters_R11_2	0.1072	0.0003
Nonpareil_PFS2	Nonpareil_ArbMarine	0.1041	0.0005
Stukey_5_2	Winters_PFS	0.1033	0.0005
Stukey_6_1	Winters_PFS	0.1033	0.0005
Nonpareil_PFS3	Nonpareil_Esparto2	0.1012	0.0007
Carmel_ArbMarine	Winters_PFS	0.0993	0.0009
Nonpareil_PFS3	Carmel_ArbMarine	0.0993	0.0009
Stukey_6_2	Nonpareil_PFS3	0.0984	0.001
Stukey_5_1	Winters_PFS	0.0966	0.0012
Nonpareil_ArbMarine	Winters_PFS	0.0963	0.0012
Nonpareil_PFS2	Mission_PFS	0.0944	0.0015
Mission_Wolfskill	Winters_R11_2	0.0933	0.0017
Stukey_5_2	Carmel_Wolfskill	0.0932	0.0018
Nonpareil_Esparto2	Carmel_PFS	0.0921	0.002
Stukey_6_2	Nonpareil_PFS1	0.092	0.002
Stukey_6_1	Carmel_ArbMarine	0.0915	0.0021
Stukey_5_1	Carmel_Wolfskill	0.0903	0.0024
Winters_PFS	Winters_R11_1	0.0891	0.0028
Nonpareil_PFS1	Carmel_PFS	0.0888	0.0029
Nonpareil_Esparto2	Carmel_ArbMarine	0.0874	0.0034
Mission_PFS	Winters_R11_2	0.0829	0.0054
Mission_Wolfskill	Carmel_Wolfskill	0.0798	0.0074
Turkmen_Repo1	Mission_PFS	0.0763	0.0104
Stukey_6_1	Carmel_Wolfskill	0.0693	0.0201
Nonpareil_PFS1	Carmel_ArbMarine	0.0676	0.0233
Nonpareil_PFS2	Winters_R11_2	0.0664	0.026
Carmel_PFS	Winters_R11_2	0.0565	0.0578
Mission_Wolfskill	Carmel_ArbMarine	0.0531	0.0746
Nonpareil_Esparto1	Mission_PFS	0.0502	0.0922
Carmel_Wolfskill	Winters_R11_1	0.0487	0.1026
Stukey_6_2	Winters_R11_2	0.0465	0.1185
Turkmen_Repo1	Winters_R11_2	0.0463	0.1201
Nonpareil_PFS2	Carmel_ArbMarine	0.0451	0.1299
Carmel_Wolfskill	Winters_Browne	0.0429	0.1501
Nonpareil_ArbMarine	Winters_R11_2	0.0413	0.1661
Nonpareil_PFS1	Nonpareil_Esparto1	0.0383	0.1985
Stukey_5_1	Nonpareil_Esparto2	0.0376	0.2066
Nonpareil_Esparto2	Winters_PFS	0.0344	0.249
Stukey_6_2	Carmel_Wolfskill	0.0298	0.3174
Nonpareil_ArbMarine	Mission_Wolfskill	0.0285	0.3393
Carmel_ArbMarine	Winters_R11_2	0.0249	0.4036
Mission_PFS	Carmel_PFS	0.0232	0.4359

Stukey_5_2	Winters_R11_2	0.0226	0.4487
Nonpareil_Esparto2	Mission_Wolfskill	0.0183	0.54
Stukey_5_2	Nonpareil_Esparto2	0.0183	0.5396
Mission_PFS	Carmel_Wolfskill	0.0113	0.7039
Nonpareil_Esparto2	Mission_PFS	0.0111	0.7101
Nonpareil_ArbMarine	Mission_PFS	0.0097	0.7443
Stukey_5_1	Winters_R11_2	0.0085	0.7761
Nonpareil_Esparto2	Winters_Browne	0.0068	0.8192
Mission_PFS	Carmel_ArbMarine	0.0051	0.8642
Stukey_6_1	Winters_R11_2	0.0046	0.8774
Nonpareil_PFS1	Nonpareil_ArbMarine	0.0014	0.9619
Winters_PFS	Winters_R11_2	-0.0004	0.9899
Nonpareil_PFS3	Nonpareil_ArbMarine	-0.001	0.9727
Nonpareil_Esparto2	Winters_R11_1	-0.0094	0.7519
Nonpareil_PFS1	Carmel_Wolfskill	-0.0194	0.5144
Nonpareil_PFS1	Winters_R11_2	-0.0249	0.4037
Stukey_6_1	Nonpareil_Esparto2	-0.0596	0.0456
Nonpareil_PFS1	Nonpareil_Esparto2	-0.0616	0.0388
Winters_R11_2	Winters_R11_1	-0.0618	0.0383
Winters_Browne	Winters_R11_2	-0.0674	0.0237
**Average**		**0.1790**	
